# Multi-Trait Index-Based Selection of Drought Tolerant Wheat: Physiological and Biochemical Profiling

**DOI:** 10.3390/plants14010035

**Published:** 2024-12-26

**Authors:** Mohammed Mohi-Ud-Din, Md. Alamgir Hossain, Md. Motiar Rohman, Md. Nesar Uddin, Md. Sabibul Haque, Mahmudul Hasan Tahery, Mirza Hasanuzzaman

**Affiliations:** 1 Department of Crop Botany, Bangabandhu Sheikh Mujibur Rahman Agricultural University, Gazipur 1706, Bangladesh; mmu074@bsmrau.edu.bd (M.M.-U.-D.);; 2 Department of Crop Botany, Bangladesh Agricultural University, Mymensingh 2202, Bangladesh; 3 Plant Breeding Division, Bangladesh Agricultural Research Institute, Gazipur 1701, Bangladesh; 4 Department of Agronomy, Faculty of Agriculture, Sher-e-Bangla Agricultural University, Dhaka 1207, Bangladesh

**Keywords:** *Triticum aestivum* L., photosynthesis, drought, osmoprotectants, hierarchical cluster analysis, plant breeding

## Abstract

Drought is a detrimental abiotic stress that severely limits wheat growth and productivity worldwide by altering several physiological processes. Thus, understanding the mechanisms of drought tolerance is essential for the selection of drought-resilient features and drought-tolerant cultivars for wheat breeding programs. This exploratory study evaluated 14 wheat genotypes (13 relatively tolerant, one susceptible) for drought endurance based on flag leaf physiological and biochemical traits during the critical grain-filling stage in the field conditions. Measurements included six physiological, seven gas exchange, six photosystem II, six stomatal, three reactive species, seven metabolomic solutes, and two biomass traits. All parameters were significantly influenced by drought, with varying genotypic responses. Hierarchical cluster analysis (HCA) categorized genotypes into three drought tolerance groups based on trait performance. Seven genotypes in Cluster 2 (BARI Gom 26, BARI Gom 33, BD-631, BD-600, BD-9910, BD-9889, BD-637) exhibited superior drought tolerance, characterized by minimal changes in physiological traits and biomass accumulation, reduced oxidative stress markers, and increased accumulation of osmoprotectants. The innovative multi-trait genotype-ideotype distance index (MGIDI) further ranked wheat genotypes in regard to drought tolerance, identifying BARI Gom 33, BARI Gom 26, BD-9889, and BD-600 as top performers. Notably, all these top-ranking genotypes belonged to Cluster 2, previously identified as the highest-performing group in the HCA. The identified genotypes with superior drought tolerance offer valuable genetic resources for enhancing wheat productivity in water-limiting environments. Traits related to photosynthetic activity, biomass gain, leaf conductance, water stress, and osmoprotection showed high selection differentials and heritability in MGIDI analysis, indicating their potential as selection targets for drought-tolerant wheat. Overall, the strategic approaches have yielded novel insights into genotype screening that can be directly applied to deepen our understanding of drought tolerance mechanisms in wheat.

## 1. Introduction

Wheat (*Triticum aestivum* L.), a staple cereal crop in global agriculture, supplies essential carbohydrates to 35% of the world’s population. However, wheat production declines worldwide are primarily the result of yield-limiting stressors, the most critical of which is drought, which poses a significant threat to global wheat supply and dietary security [1,2] Worldwide, drought stress curtails wheat production by hindering growth and altering plant physiology [3]. Drought stress has caused global wheat losses of more than 12% [4], and even a moderate water scarcity results in a 20% decrease in wheat yield [5]. Several physiological markers like leaf tissue water content (LWC) and cell membrane stability (CMS) can aid in identifying drought-tolerant wheat [6,7,8]. Additionally, canopy temperature depression (CTD) can be a crucial selection tool for drought-tolerant genotypes [9,10].

The flag leaf of wheat plant is the primary photosynthetic structure, contributing significantly to biomass production [11]. As a key process in primary metabolism, photosynthesis is highly susceptible to environmental stresses, including water scarcity [8,12]. The net photosynthesis rate (*P_n_*) of leaves is reduced during drought stress due to both stomatal and non-stomatal restrictions [13]. While stomatal closure is a major culprit behind reduced photosynthesis during drought [14,15]; the lower Rubisco activity can also play a vital role [16,17]. Plants constrict stomata to conserve water during drought, reducing CO_2_ intake and *P_n_* [18]. This helps plants with water use efficiency (WUE) but comes at the cost of lower *P_n_* [19,20]. Drought further weakens *P_n_* by limiting CO_2_ diffusion within the leaf and reducing Rubisco activity and electron transport [21]. Leaf gas exchange measurements, including *P_n_*, stomatal and mesophyll conductance, rate of transpiration, intercellular CO_2_ concentration (*C_i_*), and water use efficiency (WUE) are used to assess drought tolerance in crops [14].

Pulsed-modulated chlorophyll fluorometry is a rapid, non-destructive technique to assess plant photosynthetic activity under stress [18], and is particularly useful for high-throughput studies [22]. This method measures chlorophyll *a* fluorescence (ChlF), which reflects the health of photosystem II (PSII), the most stress-sensitive part of photosynthesis [1,12]. Drought disrupts PSII function of plants, including wheat, leading to a reduction in maximum photochemical efficiency of PSII (*F_v_*/*F_m_*), maximum energy conversion efficiency of PSII (*F_v_*/*F_o_*), quantum yield of PSII (Φ_PSII_), photochemical quenching (qP), and electron transport rate (ETR), while increasing non-photochemical quenching (NPQ) and other photosynthetic parameters [23,24,25,26,27,28,29,30].

Drought limits carbon absorption, creating a disparity between electron excitation and utilization via photosynthesis. This imbalance leads to the increased generation of reactive oxygen species (ROS) like superoxide radicals (O_2_^•−^) and hydrogen peroxide (H_2_O_2_), inducing oxidative stress [31,32]. These ROS damage cell membranes, raising malondialdehyde (MDA) levels indicating the extent of oxidative stress in plants [33,34]. Additionally, drought can cause the production of toxic aldehydes like methylglyoxal (MG), which disrupts cellular function, hindering growth and development [35].

Plants combat drought by accumulating solutes like proline, ornithine, glycene betaine, trehalose, fructose, sucrose, and total soluble sugar in their cells [36]. These solutes act like tiny sponges, maintaining cell turgor and reducing water loss [37]. Measuring levels of these solutes can even help identify drought-tolerant varieties [38]. Proline and glycine betaine also offer additional benefits: they protect against cellular damage, stabilize proteins, and even serve as alternative nitrogen and carbon sources under drought conditions [39]. Drought can also trigger the accumulation of sugars and sugar alcohols [40]. These sugars help maintain the structure and function of proteins during water stress [41]. Overall, these various solutes play a complex balancing role within plant cells, helping them maintain osmotic balance in plants and thus turgor pressure to endure photosynthesis, and enable plants to survive under drought stress [42,43,44].

Multivariate analyses, particularly hierarchical cluster analysis, principal component analysis, and linear discriminant analysis, have been frequently utilized in breeding to reduce variable dimensionality, assess genetic diversity, and identify drought-tolerant varieties [6,45]. While these methods effectively reveal trait relationships, ranking genotypes depending on diverse traits remains a challenge [46,47]. The innovative multi-trait genotype-ideotype distance index (MGIDI) approach can overcome this challenge and serves as a strategic tool for genotype ranking based on multiple traits with higher genetic differentials [47,48]. This study employed MGIDI to rank wheat genotypes based on physiological multivariate data and facilitate the reliable selection of drought-tolerant genotypes.

Genetic diversity is essential for developing improved crop varieties with enhanced adaptability to changing environments [49,50]. However, limited understanding of drought tolerance mechanisms, complex plant responses, and genotype-environment interactions hinders breeding progress [51]. Integrating diverse data on morphology, physiology, and gene expression is crucial for effectively selecting drought-tolerant wheat genotypes. In light of these considerations, this study aimed to evaluate 14 local wheat genotypes under drought conditions at the rapid grain-filling stage, focusing on a range of physiological, gas exchange, photochemical, stomatal, biochemical, and yield-related traits, and their association with drought resistance. The study also explored whether such multidimensional and multi-trait data could be efficiently handled using the MGIDI in combination with multivariate analyses to accurately predict drought resistance mechanisms. Traits with high heritability, positive correlations with yield, and significant selection differentials are expected to help identify promising donor genotypes for breeding programs. The findings of this work have the potential to support the development of drought-resistant wheat suited for water-limited environments.

## 2. Materials and Methods

### 2.1. Plant Materials and Growing Conditions

A total of 14 wheat genotypes were used in the experiment, which were selected from a prior screening for drought tolerance using spectral reflectance indices and yield traits [52]. Among them, 13 genotypes (BD-466, BD-477, BD-525, BD-600, BD-631, BD-637, BD-666, BD-9889, BD-9910, AS-10632, BARI Gom 26, BARI Gom 33, and BAW1-1147) were observed to be relatively tolerant in the field study, while AS-10617 showed susceptibility to drought stress. Table S1 provides more information regarding the selected genotypes.

The experiment was performed at the field laboratory of the department of Crop Botany, Bangabandhu Sheikh Mujibur Rahman Agricultural University (24.038° N, 90.397° E), Gazipur, Bangladesh. The climate in this area is under the typical subtropical monsoon. Bread wheat is typically sown in the study region between mid-November and mid-December. The field soil texture was silt loam with 26% sand, 50% silt, and 24% clay). The full field capacity (FC) of soil was attained at 30.6% volumetric soil water content. The physical and chemical properties of the experimental soil were presented in Table S2. Monthly meteorological data of the wheat growing season, with the averaged data from the past ten years at the study area, are presented in Table S3.

### 2.2. Experimental Design and Drought Imposition

A two-factor randomized complete block design was followed with three replications. Seeds of 14 wheat genotypes were sown in the middle of November. The seeds were disinfected with a commercial fungicide that contained carboxin and thiram before the seed sowing. All agronomic practices were done as per the recommendation. Throughout the cropping season, the wheat plants were irrigated five times at 20, 45, 60, 70, and 80 DAS in control plots. In contrast, only the first two irrigations (20 and 45 DAS) were applied in drought-treated plots to support vegetative growth, after which irrigation was discontinued. In each irrigation, about 4.35 cm of water was supplied to each plot.

### 2.3. Determination of Crop Phenology and Soil Moisture Content

**Figure 1:**
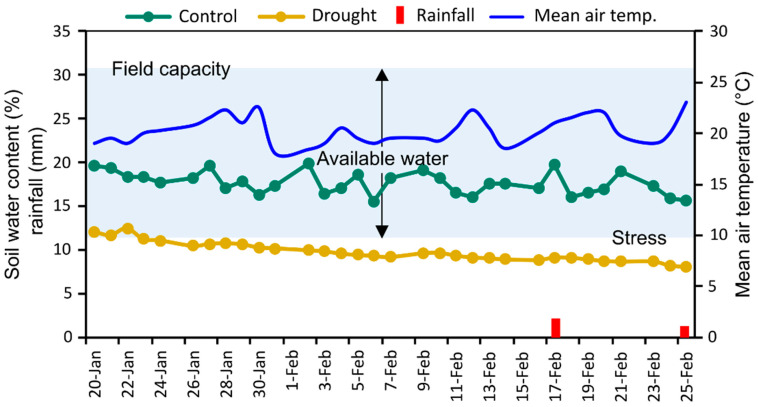
Mean air temperature, rainfall, and soil moisture content (%) in each drought-simulated and control plot during the reproductive phases of wheat genotypes. The experimental site’s soil type is silt loam, with silt, sand, and clay contributions of 50, 26, and 24%, respectively. The full field capacity of the soil was attained at a volumetric soil moisture content of 30.6%. The blue shading area indicating arrows show the available water for plant. Every day, the percentage of soil water in the randomly chosen control and drought-treated plots (*n* = 15) was assessed.

The heading (GS55) and the anthesis (GS65) of the genotypes were recognized when the spikes were visible, and 50% of the spikes started flowering, respectively [53]. For the subsequent measurements at 14 DAA, a schedule was made at the time of anthesis for each genotype as the phenological events varied between genotypes. A digital soil moisture content meter (PMS-714, Lutron Electronic Enterprise Co., Ltd., Taipei, Taiwan) was used to record the percentage of soil water at a depth of 15 cm in per day from fifteen randomly selected each of the control and drought-treated plots. Figure 1 shows average daily soil water content data. The FC of the plot soil was estimated at the start of the experiment following the method of Ogbaga et al. [54].

### 2.4. Leaf Relative Water Content Measurement

Leaf relative water content (LRWC) was determined following the method of Mohi-Ud-Din et al. [6]. Briefly, a 0.5 g leaf sample (FW) was dipped in 100 mL of distilled water for a duration of 4 h. After weighing the turgid leaf samples (TW), they were oven-dried for 48 h at 80 °C, and then the samples’ dry weights (DW) were noted. The process was repeated three times for each replicate.

$$LRWC\;\left(\%\right)=\frac{FW-DW}{TW-DW}\times 100$$

### 2.5. Excised Leaf Water Retention (ELWR) and Relative Water Loss (RWL) Determination

Excised leaf water retention (ELWR) and relative water loss (RWL) were measured as described by Geravandi et al. [55]. In brief, five flag leaves from each plot under control and drought conditions were collected at the GS75 stage. The flag leaf samples were weighed immediately (FW) and allowed to wilt for 4 h at 35 °C; the weight of the wilted leaves (WW) was recorded and then oven-dried for 48 h at 80 °C to obtain dry weight (DW). The ELWR and RWL were then calculated using the following formula:

$$ELWR\;\left(\%\right)=\left(1-\frac{FW-WW}{FW}\right)\times 100$$

$$ELWR\;\left(\%\right)=\left(1-\frac{FW-WW}{FW}\right)\times 100$$

### 2.6. Leaf Greenness and Canopy Temperature Depression Measurement

The leaf greenness and canopy temperature depression were recorded every day from heading (GS55) to physiological maturity (GS90). The leaf greenness of ten main shoot flag leaves from each plot was determined using a portable Soil Plant Analysis Development (SPAD) meter (SPAD-502, Konica Minolta Inc., Osaka, Japan) index.

The canopy temperature (CT) and ambient air (AT) were measured between 11.30 a.m. and 12.30 p.m. by a hand-held infrared thermometer and the instant air temperature was recorded by connecting a sensor with the device (IR-818, URCERI, Highland, CA, USA; the distance-spot ratio of 13:1). The spotted canopy, approximately one meter from the thermometer, was reached at an angle of approximately 30 degrees to the horizontal. From each plot, 10 records were captured and mean data were used for analysis. Following the Fischer et al. [56] approach, canopy temperature depression (CTD) was computed using the following formula:

CTD=AT−CT

### 2.7. Determination of Cell Membrane Stability

Cell membrane stability (CMS) was estimated following the method of Sairam et al. [57]. Briefly, 30 leaf discs with 0.7 cm in diameter (two sets) were cut from three flag leaves and put in glass tubes filled with 10 mL of distilled water. The leaf discs were divided into two sets—one set was incubated at 40 °C for 30 min in a water bath and the other set at 100 °C for 15 min. Then the electrical conductivities (EC) were measured using an EC meter (EC-400L, Human Lab Instrument Co., Suwon, Republic of Korea). The procedure was carried out three times in each replication. CMS was computed using the following formula:

$$CMS\left(\%\right)=\left(1-\frac{C_{1}}{C_{2}}\right)\times 100$$

### 2.8. Gas Exchange Measurements

At the GS75 stage, gas exchange parameters were measured from the middle portion of flag leaves from each plot between 10:30 am and 11:30 am using a hand-held photosynthetic system (LI-6400XT, Li-COR Environmental, Lincoln, NE, USA). Three intact flag leaves of the main shoots from each replication per growing condition were selected to measure gas exchange parameters. The leaves were completely exposed and aligned normally to the receiving solar radiation during the measurement to ensure the maximum possible photosynthetic photon flux density (approximately 1200 µmol m^−2^ s^−1^). After a 5-min acclimation period in the leaf chamber, gas exchange measurements were conducted at an atmospheric CO_2_ concentration of 370 ± 75 μmol mol^−1^. The rate of photosynthesis (*P_n_*), transpiration rate (*T_r_*), stomatal conductance (*g_s_*), intracellular CO_2_ concentration (*C_i_*), and leaf-to-air vapor pressure deficit (VPD_LA_) were recorded. The apparent mesophyll conductance (*g_m_*) was determined as *P_n_*/*C_i_* [58]. The leaf-level instantaneous water use efficiency (WUE_L_) was computed as *P_n_*/*T_r_* [59].

### 2.9. Measurement of Chlorophyll a Fluorescence

After performing gas exchange measurements, intact flag leaves were dark-adapted for 30 min (using aluminum foil paper) before being measured chlorophyll fluorescence (ChlF) parameters with a Junior-PAM chlorophyll fluorometer (Heinz Walz, Germany) as reported by Khan et al. [60]. Specifically, the minimal fluorescence (*F_o_*) and maximum fluorescence (*F_m_*) of dark-adapted leaves were measured using a low measuring beam at a low light intensity of 125 µmol m^−2^ s^−1^. On the other hand, under light-adapted conditions, minimal and maximum fluorescence (*F′_o_* and *F′_m_*, respectively) were measured in the same leaves with a saturating light of 720 µmol m^−2^ s^−1^, as well as steady-state fluorescence (*F_s_*). Then the ChlF parameters such as maximal quantum efficiency of PSII (*F_v_*/*F_m_*), maximum energy conversion potential of PSII (*F_v_*/*F_o_*), quantum yield of PSII (Φ_PSII_), qP, NPQ, and electron transport rate (ETR) were computed using the methods described by [61,62,63].

### 2.10. Stomatal Measurements and Analysis

At the GS75 stage, clear nail polish was used to capture stomatal imprints from the upper and lower surfaces of five intact flag leaves per replication. The delicate film was carefully peeled off and gently laid on a glass slide, wetted with a drop of water for improved visibility. Stomatal density (SD) was calculated by counting the total number of stomata in ten randomly selected, non-overlapping quadrate fields of view (2.38 mm^2^) per leaf at ×100 magnification using a light microscope (AxioCam, Carl Zeiss Microscopy GmbH, Jena, Germany). Stomatal size (SS) and aperture space (AS) were calculated from ten randomly selected stomata per field of view by multiplying the length by the width of the guard cell and stomatal pore, respectively using ZEN lite 2012 software. All parameters were reported as the average of the adaxial and abaxial leaf surfaces.

Specific stomatal conductance (S*g_s_*) was calculated by dividing *g_s_* by SD [64]. The stomatal area index (SAI), an integrative stomatal metric, was derived by multiplying SD by SS and represented in mm^2^ stomata mm^−2^ leaf [30]. Another integrative parameter, maximum stomatal diffusive conductance (*g_s_*_.max_) was computed according to Franks and Beerling [65]:

$$g_{s.max}=\frac{d\;.\;SD\;.\;a_{max}}{1.6v\left(l+\frac{\pi }{2}\sqrt{a_{max}/\pi }\right)}$$

where, *d* is the water vapor diffusivity in air at 25 °C = 2.82 × 10^−5^ m^2^ s^−1^, SD and *a*_max_ are the stomatal density and maximum area of the open stomatal pore, respectively and calculated as *π*(*p*/2)^2^ where *p* is the pore length of stomata, *v* is the air molar volume at 25 °C = 24.5 m^3^ mol^−1^, and *l* is the pore depth of fully open stoma and equals to W/2 where W is the width of the guard cell.

### 2.11. Malondialdehyde (MDA), Hydrogen Peroxide (H_2_O_2_) and Methylglyoxal (MG) Contents

The MDA and H_2_O_2_ were extracted from 0.5 g fresh flag leaf tissues by homogenizing in 3 mL of 5% (*w*/*v*) trichloroacetic acid (TCA). The homogenate was centrifuged at 11,500× *g* for 10 min, and the supernatants were collected for the assays. MDA content was determined using thiobarbituric acid (TBA) as the reactive material according to the protocol of Heath and Packer [66] using the extinction coefficient of 155 mM^−1^cm^−1^ and expressed as nmol g^−1^ FW. The H_2_O_2_ content was measured spectrophotometrically according to the procedure of Yang et al. [67]. The H_2_O_2_ concentration was calculated using the extinction coefficient of 0.28 μM^−1^ cm^−1^ and reported as μmol g^−1^ FW. Methylglyoxal (MG) was extracted from the flag leaves and the samples were prepared and neutralize according to the procedure of Yadav et al. [68]. MG was estimated spectrophotometrically using *N*-acetyl-l-cysteine assay according to the method described by Wild et al. [69]. MG content was calculated from the sample absorbances using a standard curve of known concentrations and expressed as µmol g^−1^ FW.

### 2.12. Metabolomic Solutes and Osmolytes Estimation

The flag leaves proline (Pro) content was quantified using spectrophotometry following the acid-ninhydrin assay and was calculated as μmol g^−1^ FW [70]. Ornithine (Orn) content (μmol g^−1^ FW) of the flag leaves was estimated spectrophotometrically following the procedure of Gaitonde [71] with the help of a standard curve. Glycine betaine (GB) was estimated spectrophotometrically following 1,2-dichloroethane procedure, as described by Valadez-Bustos et al. [72], and expressed as μmol g^−1^ FW using a standard curve. Total free amino acid (TFAA) content was assayed spectrophotometrically as the procedure of Lee and Takahashi [73]. TFAA content was calculated from a standard curve created with a series of known concentration of glycine. TFAA content was reported as mg g^−1^ FW. Sugar content from the flag leaf (0.2 g) was extracted in 2 mL of 80% (*v*/*v*) ethanol by boiling for 5 min. The procedure was repeated thrice. The decant was then centrifuged at 11,500× *g* for 15 min, and ethanol was evaporated from the supernatant in a water bath at 80 °C. The residue was eluted in 5 mL of deionized water and stored at 4 °C. This supernatant was used for the spectrophotometric assays of trehalose (Tre), sucrose (Suc), and total soluble sugar (TSS). Trehalose (Tre) content was determined following the procedure of Li et al. [74] using a standard curve and expressed as μmol g^−1^ FW. Sucrose content in the flag leaf was measured following the protocol explained by van Handel [75] and the Suc concentration was calculated following the standard curve, and reported as mg g^−1^ FW. Flag leaf TSS concentration was determined according to Malik and Srivastava [76]. The amount of TSS was quantified using the standard curve and reported as mg g^−1^ FW.

### 2.13. Statistical Analysis

The means of the daily SPAD and CTD values along with all other studied traits, fitted to a linear mixed model for the factorial RCBD in R-v4.1.0 [77]. The following equation was used to run the analysis of variance (ANOVA) in order to ascertain whether the parameters were significantly impacted by genotype, growth condition, and their interactions:

$$Y_{ijk}=\mu +\alpha_{i}+\beta_{j}+\gamma_{k(j)}+{\left(\alpha \beta \right)}_{ij}+\varepsilon_{ijk}$$

where *Y_ijk_* is observation of the responsible variables (genotype, growth conditions and block); *µ* is the overall population mean impact; *α_i_* and *β_j_* are the impact of the *i*-th genotype and *j*-th growing conditions, respectively; γ*_k_*_(*j*)_ is the impact of the *k*-th replication within the *j*-th growing conditions; (*αβ*)*_ij_* is the interaction effect between genotype and growth conditions; and *ε_ijk_* is the random or unexplained error associated with the *i*-th genotype, *j*-th growing conditions and *k*-th replication. This error is assumed to be normally and independently distributed with a mean of zero and a constant variance, σ^2^*_ε_*.

The variations between the treatment means were compared using the paired *t*-test and illustrated in boxplots with the help of ggplot2 and ggpubr packages of R program [78]. Lollipop plot was created using R packages—ggplot2 along with reshape2 [78]. The ggplot2 and ggpubr packages were also used to calculate linear correlation and to create scatterplots [78]. Using the relative trait values (ratio of trait_drought_ to trait_control_), hierarchical clustering was performed. Heatmap with hierarchical clusters (using Euclidean distance and wardD2 method) were generated by the package pheatmap of R [79].

Using the R package metan, genotype means were rescaled and subjected to factor analysis. The MGIDI was then computed as the Euclidean distance between the factor scores of each genotype and the ideal genotype, following the procedure outlined by Olivoto et al. [47] as-

$${MGIDI}_{i}={\left[\sum_{j=1}^{f}{\left(\gamma_{ij}-\gamma_{j}\right)}^{2}\right]}^{0.5}$$

where *MGIDI_i_* represents the MGIDI index for the genotype *i*; γ*_ij_* denotes the score of the genotype *i* in the factor *j* (*i* = 1, 2, …, *t*; *j* = 1, 2, …, *f*), where *t* and *f* denote the genotype and factor numbers, respectively; and γ*_j_* is the *j*-th score for the ideal genotype. A genotype with a lower MGIDI is closer to the ideotype and, therefore, possesses the desired characteristics for all the traits examined.

Genotype strengths and weaknesses were determined by calculating the extent between every genotype and the ideal genotype for each factor. Factors with smaller distances to the ideal genotype indicate traits closer to the desired phenotype. To visualize trait relationships and their association with the genotypes, principal component analysis (PCA) was conducted with genotypes as observations and traits as variables. A PCA-biplot was generated using the fviz_pca_biplot() function belongs to the factoextra library in the R program [80].

## 3. Results

### 3.1. Analysis of Variance

All the traits were substantially affected by both of the factors (14 wheat genotypes and two growing conditions) but with diverse magnitudes (Table S4). The main effects of genotype and growing condition in the analysis of variance (ANOVA) were highly significant for all physiological traits, gas exchange parameters, stomatal characteristics, (ChlF) parameters, prooxidants, osmolytes, and yields. A modest number of traits showed significant genotype × growth condition interaction effects, but the contribution to the variation was lesser than the main effects. The block (replication) effect was nonsignificant for all the studied traits.

### 3.2. Impact of Soil Drought on the Measured Traits

All the studied traits substantially altered under drought condition compared to control (Figures S1 and S2). Among them, physiological traits, stomatal characteristics, and yield traits decreased significantly due to drought, while all the prooxidants and osmolyte contents increased significantly under drought conditions. Among the gas exchange parameters, *P_n_*, *g_s_*, *g_m_*, and *T_r_* decreased significantly, while a marked increase in *C_i_*, VPD_LA_, and instantaneous WUE_L_ was recorded under drought. However, except NPQ, all the chlorophyll fluorescence parameters decreased significantly under drought. The descriptive statistics, viz., range, mean, median, quartiles of the traits studied under both growing conditions, and their significance level were presented in Figures S1 and S2. The genotypic response of the investigated traits to drought is presented in the following heads.

#### 3.2.1. Physiological Traits

**Figure 2:**
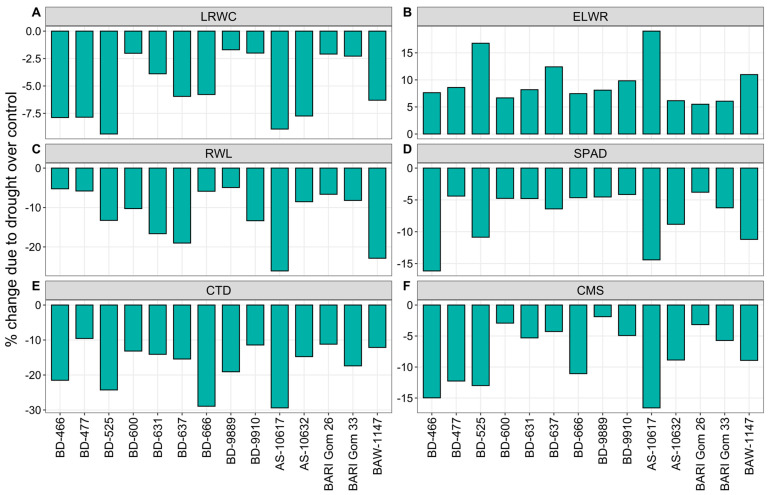
Percent change in (**A**) leaf relative water content (LRWC), (**B**) excised leaf water retention (ELWR), (**C**) relative water loss (RWL), (**D**) chlorophyll index (SPAD), (**E**) canopy temperature depression (CTD), and (**F**) cell membrane stability (CMS) of 14 wheat genotypes under drought stress relative to well-watered controls.

Among the physiological traits, leaf relative water content (LRWC), relative water loss (RWL), chlorophyll index (SPAD), canopy temperature depression (CTD), and cell membrane stability (CMS) were decreased significantly due to drought, while the excised leaf water retention (ELWR) increased markedly under drought in all wheat genotypes (Figure 2 and Figure S3). The highest decrease (9%) in LRWC was recorded in BD-525 and AS-10617 due to drought, and the lowest (2%) in BD-600, BD-9889, BD-9910, BARI Gom 26, and BARI Gom 33 (Figure 2A). Under drought, the highest 17% increase in the ELWR was found in the genotype BD-525, while the lowest (6%) increase in AS-10632, BARI Gom 26, and BARI Gom 33 (Figure 2B). The highest (26%) decrease in RWL showed by AS-10617, whereas genotypes BD-466 and BD-9889 exhibited the lowest (5%) decrease under drought (Figure 2C).

Compared to the control, the highest 16% decrease in flag leaf chlorophyll index (SPAD) was recorded in BD-466, and the lowest 4% in BD-477, BD-9910, and BARI Gom 26 under drought stress (Figure 2D). Drought stress resulted in the highest 29% decrease in CTD of the genotypes BD-666 and AS-10617, while the lowest (10%) in BD-477 (Figure 2E). Due to drought, the highest (17%) decrease in CMS was recorded in AS-10617, however, the lowest (2%) was in BD-9889 (Figure 2F).

#### 3.2.2. Gas Exchange Parameters

**Figure 3:**
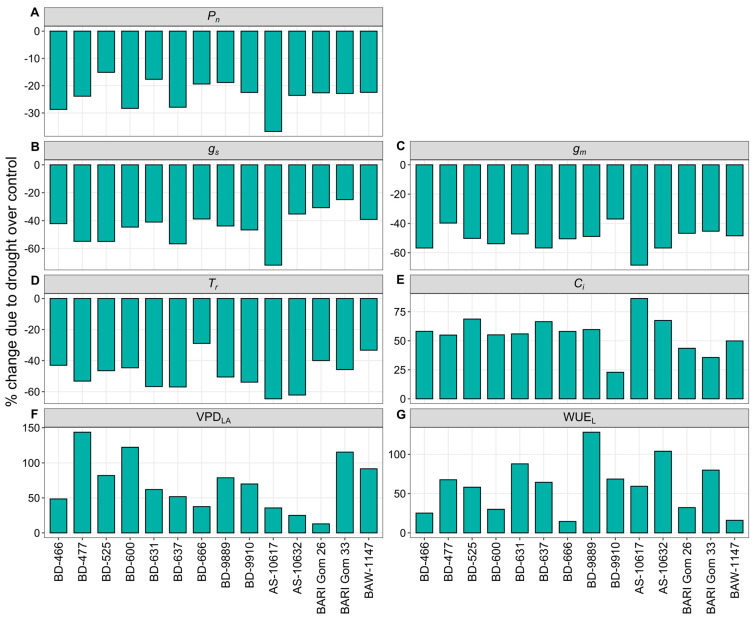
Percent change in the gas exchange parameters such as (**A**) Net photosynthesis rate (*P_n_*), (**B**) stomatal conductance (*g_s_*), (**C**) apparent mesophyll conductance (*g_m_*), (**D**) transpiration rate (*T_r_*), (**E**) intercellular CO_2_ concentration (*C_i_*), (**F**) leaf-to-air vapor pressure deficit (VPD_LA_), and (**G**) leaf-level instantaneous water use efficiency (WUE_L_) of 14 wheat genotypes under drought stress relative to well-watered controls.

Drought stress considerably reduced net *P_n_*, *g_s_*, *g_m_*, and *T_r_*, but it significantly increased intercellular carbon dioxide (*C_i_*), leaf-to-air vapor pressure deficit (VPD_LA_), and leaf-level water use efficiency (WUE_L_) in all wheat genotypes (Figure 3 and Figure S4). Due to drought, genotype AS-10617 had the highest declines in *P_n_*, *g_s_*, *g_m_*, and *T_r_*, with drops of 37, 72, 68, and 65%, respectively, whereas BD-525, BARI Gom 33, BD-9910, and BD-666 had the lowest declines of 15, 25, 37, and 29% (Figure 3A–D). Drought stress caused the highest increases in *C_i_*, VPD_LA_, and WUE_L_ in genotypes AS-10617, BD-477, and BD-9889, with rises of 86, 144, and 128%, respectively, whereas BARI Gom 33, BARI Gom 26, and BD-666 had the least increases of 36, 13, and 15% (Figure 3E–G).

#### 3.2.3. Chlorophyll *a* Fluorescence Parameters

**Figure 4:**
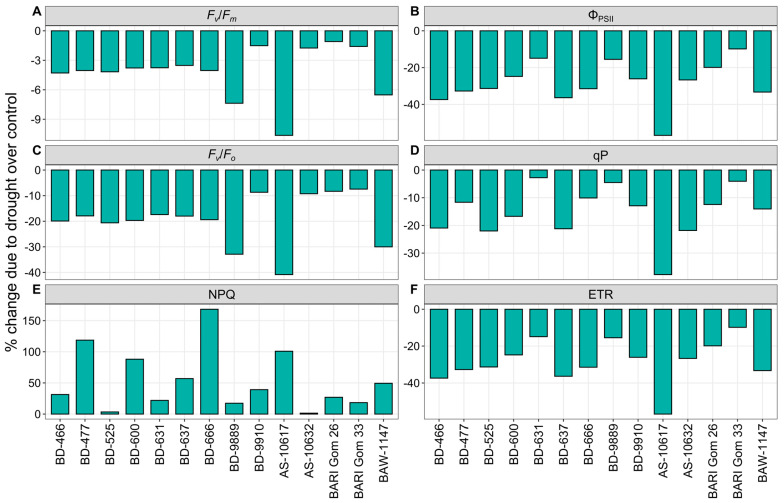
Percent change in chlorophyll *a* fluorescence parameters—(**A**) maximal quantum efficiency of PSII (*F_v_*/*F_m_*), (**B**) quantum yield of PSII (Φ_PSII_), (**C**) maximum energy conversion potential of PSII (*F_v_*/*F_o_*), (**D**) photochemical quenching (qP), (**E**) non-photochemical quenching (NPQ), and (**F**) electron transport rate (ETR) of 14 wheat genotypes under drought stress relative to well-watered controls.

Under drought stress, maximal quantum efficiency of PSII (*F_v_*/*F_m_*), quantum yield of PSII (Φ_PSII_), maximum energy conversion potential of PSII (*F_v_*/*F_o_*), qP, and electron transport rate (ETR) were decreased significantly, while NPQ increased significantly in all wheat genotypes (Figure 4 and Figure S5). Genotype AS-10617 exhibited the highest decreases of 11, 57, and 41% in *F_v_*/*F_m_*, Φ_PSII_, and *F_v_*/*F_o_*, respectively, due to drought (Figure 4A–C). The lowest decrease in *F_v_*/*F_m_* (1%) was recorded in the genotypes BARI Gom 26, but the genotypes BARI Gom 33 showed the lowest reduction in Φ_PSII_ (10%) and *F_v_*/*F_o_* (7%) (Figure 4A–C). Drought stress resulted in the highest declines in qP and ETR, with reductions of 38 and 57% in genotype AS-10617, respectively, whereas genotypes BD-631 and BARI Gom 33 had the lowest declines (3 and 10%) (Figure 4D,F). The highest increase (168%) in the NPQ was recorded in genotype BD-666 under drought, while the least (2%) in AS-10632 (Figure 4E).

#### 3.2.4. Stomatal Characteristics

**Figure 5:**
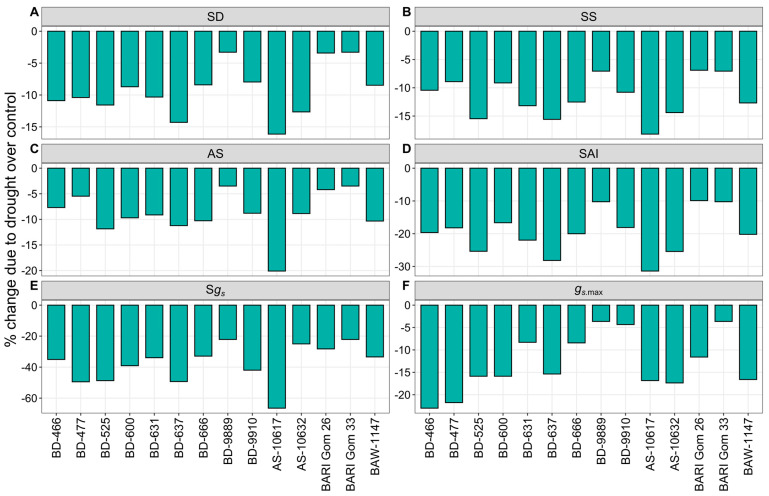
Percent change in stomatal characteristics—(**A**) stomatal density (SD), (**B**) stomatal size (SS), (**C**) aperture space (AS), (**D**) stomatal area index (SAI), (**E**) specific stomatal conductance (S*g_s_*), and (**F**) maximum stomatal diffusive conductance (*g_s_*_.max_) of 14 wheat genotypes under drought stress relative to well-watered controls.

Flag leaf stomata showed variability among different wheat genotypes and growing conditions (Figure S6). All the stomatal characteristics, viz. stomatal density (SD), stomatal size (SS), aperture space (AS), stomatal area index (SAI), specific stomatal conductance (S*g_s_*), and maximum stomatal diffusive conductance (*g_s_*_.max_), were significantly decreased under drought stress, irrespective of wheat genotypes (Figure 5 and Figure S7). Drought stress resulted in the highest decreases in SD, SS, AS, SAI, and S*g_s_* in genotype AS-10617, with declines of 16, 18, 20, 31, and 66%, respectively, whereas genotypes BD-9889, BARI Gom 26, and BARI Gom 33 showed the lowest decreases of 3, 7, 3, 10, and 22% (Figure 5A–E). The highest decline (23%) in *g_s_*_.max_ was recorded in genotype BD-466 due to drought, while the least drop of 4% was found in genotypes BD-9889, BD-9910, and BARI Gom 33 (Figure 5F).

#### 3.2.5. Reactive Oxygen Species and Osmolytes

**Figure 6:**
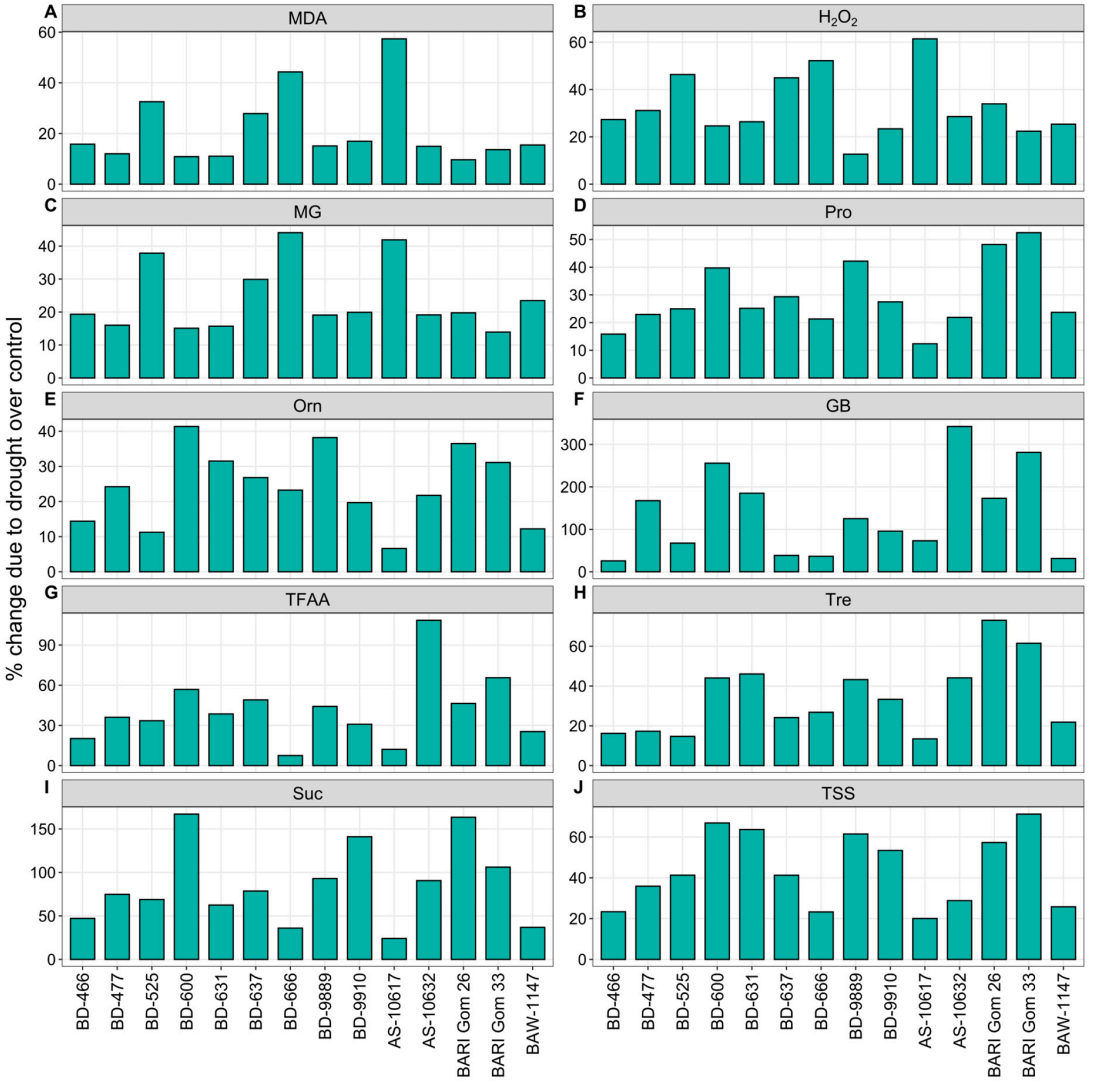
Percent change in (**A**) malondialdehyde (MDA), (**B**) hydrogen peroxide (H_2_O_2_), (**C**) methylglyoxal (MG), (**D**) proline (Pro), (**E**) ornithine (Orn), (**F**) glycine betaine (GB), (**G**) total free amino acid (TFAA), (**H**) trehalose (Tre), (**I**) sucrose (Suc), and (**J**) total soluble sugar (TSS) of 14 wheat genotypes under drought stress relative to well-watered controls.

The malondialdehyde (MDA), hydrogen peroxide (H_2_O_2_), and methylglyoxal (MG) content increased significantly during drought (Figure 6 and Figure S8). Drought stress resulted in the highest declines in MDA, H_2_O_2_, and MG contents, with reductions of 57 and 61% in genotype AS-10617, and 44% in genotype BD-666, respectively, whereas genotypes BARI Gom 26, BD-9889, and BARI Gom 33 had the lowest declines (10, 11, and 14%) (Figure 6A–C). Drought stress significantly increased the accumulation of osmolytes, namely, Pro, Orn, GB, TFAA, Tre, Suc, and TSS, contents in the wheat flag leaf of all genotypes (Figure 6 and Figure S8). Under drought, the genotypes BARI Gom 33 and BD-600 had the highest increase in Pro (52%) and Orn (41%) contents, respectively, whereas the genotype AS-10617 had the lowest (12 and 7%) (Figure 6A,B).

Genotype AS-10632 exhibited the highest increases of 342 and 108% in GB and TFAA under drought, but the genotypes BD-466 and BD-666 showed the lowest increases (26 and 8%) (Figure 6C,D). Under drought, Tre, Suc, and TSS contents increased to the highest (73, 167, and 71%) in the genotypes BARI Gom 26, BD-600, and BARI Gom 33, respectively, while the lowest increases (13, 24, and 20%) were recorded in the genotype AS-10617 (Figure 6E–G). The highest increase (153%) in Man content was exhibited by the genotype AS-10617 in response to drought, but the minimum increase (14%) was found in BARI Gom 26 (Figure 6H).

#### 3.2.6. Biological and Gain Yield

**Figure 7:**
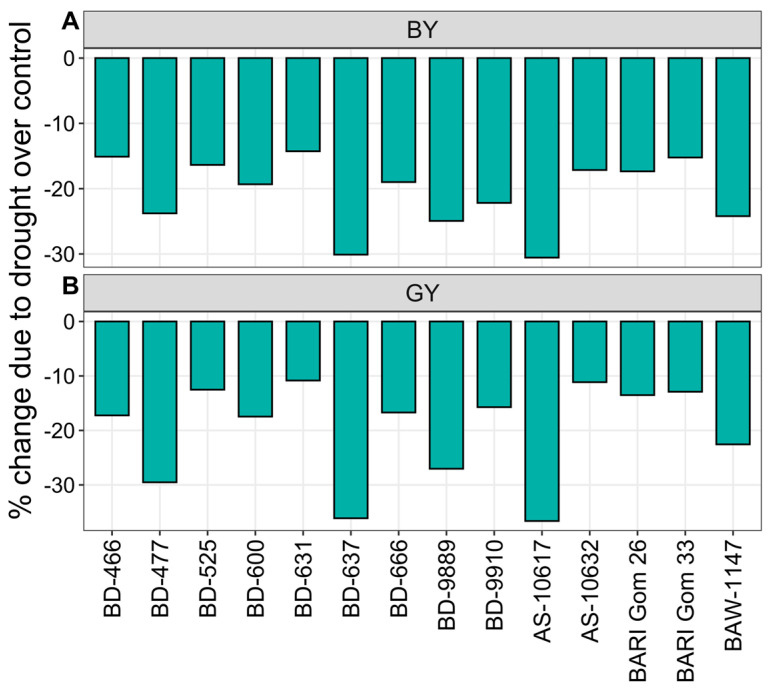
Percent change in (**A**) biological yield (BY), and (**B**) grain yield (GY) of 14 wheat genotypes under drought stress relative to well-watered controls.

Biological yield (BY) and grain yield (GY) decreased significantly under drought, irrespective of wheat genotypes (Figure 7 and Figure S9). The genotype AS-10617 exhibited the highest decreases in BY and GY in response to drought, with reductions of 31 and 37%, respectively (Figure 7). The lowest decrease in BY was recorded in the genotype BD-631 (14%), followed by BARI Gom 33 (15%) and BD-466 (16%) (Figure 7A). The genotypes BD-631 and AS-10632 showed the lowest reduction (11%) in GY, followed by BARI Gom 33 (13%) and BARI Gom 26 (14%) (Figure 7B).

### 3.3. Correlation Among Gas Exchange, Stomatal, and Fluorescence Parameters

The SPAD Chl index, *g_s_*, *g_m_*, and *T_r_* displayed a significant (*p* < 0.05) positive correlation with *P_n_* under control and drought stress, while *C_i_*, VPD_LA_, and WUE_L_ were negatively associated with *P_n_* (Figure S10). SD, AS, and S*g_s_* were positively linked with *P_n_*, with stronger relationships under drought conditions (Figure S10).

Among the fluorescence parameters, *F_v_*/*F_m_*, Φ_PSII_, *F_v_*/*F_o_*, qP, and ETR were positively and significantly correlated with *P_n_*, while NPQ showed a negative significant correlation during drought stress (Figure S10). The association between SS and SD was inverse and significant under control and drought stress, however, SAI and *g_s._*_max_ exhibited significant positive correlations with SD (Figure S11).

The SD, AS, and integrative stomatal parameters (SAI, S*g_s_*, and *g_s._*_max_) were positively and significantly associated (*p* < 0.05) with *g_s_* under drought, while SS showed a non-significant relation with *g_s_* under both growing environments (Figure S11). The BY and GY were positively correlated with LRWC, CTD, CMS, *P_n_*, *g_s_*, *F_v_*/*F_m_*, Φ_PSII_, SD, AS, Pro, and GB under both control and drought stress conditions, however, the associations were stronger and significant (*p* < 0.05) during drought (Table 1). Conversely, H_2_O_2_ exhibited significant negative correlations (*p* < 0.05) with BY and GY under water-limiting conditions (Table 1).

### 3.4. Classification of Wheat Genotypes

**Figure 8:**
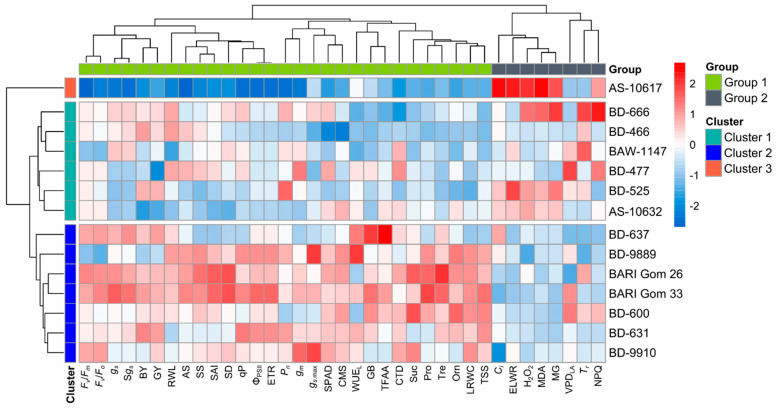
Hierarchical clustering (method = wardD2 and distance = Euclidean) and heatmap of 14 wheat genotypes versus studied traits using rescaled relative values with a scale ranged from −2 to 2. Cluster 2 genotypes displayed a greater extent of drought tolerance showing more reddish boxes followed by clusters 1 and 3 genotypes. Additional details are presented in Figure 3, Figure 4, Figure 5, Figure 6 and Figure 7.

Hierarchical cluster analysis (method = wardD2 and distance = Euclidean) was employed to categorize wheat genotypes into distinct clusters on the basis of their dissimilarity in drought tolerance potential using the relative values of the studied traits (Figure 8). Based on the genotypes’ variability in drought tolerance potential, genotypes were classified into three clusters, whereas the traits were categorized into two groups and presented as a two-way hierarchical clustering heatmap (Figure 8). The traits ELWR, *C_i_*, MDA, H_2_O_2_, MG, VPD_LA_, *T_r_*, and NPQ together formed Group 2, and the remaining traits were set in Group 1. At the genotype level, Cluster 2 has the most wheat genotypes (7), followed by Cluster 1 (6), while Cluster 3 includes only a single genotype (Figure 8). In general, the traits of Groups 1 and 2 determined Clusters 2 and 3, respectively, whereas Cluster 1 genotypes were represented by a diverse pattern in the contribution of the traits of both groups (Figure 8).

### 3.5. Principal Component Analysis and Cross-Validation Using Linear Discriminant Analysis

Principal component analysis (PCA) indicated a significant variation of bread wheat genotypes (Figure S12). There were thirteen principal components (PCs) found in total, with seven PCs with eigenvalues larger than one deemed significant. The first seven major components accounted for 90% of overall variability, and the goodness of fit for the first two presented PCs was 61.2% (Figure S12, Table S5). In the PCA-biplot, PC1 explained approximately 50.5% of the variability and was contributed by the majority of the tested traits, with the exception of VPD_LA_, WUE_L_, *T_r_*, NPQ, and TFAA whereas, the second PC accounted for around 10.7% variability and was mostly influenced by *T_r_*, WUE_L_, GB, TFAA, TSS, BY and GY (Figure S12, Table S5). Intriguingly, the PCA analysis produced clustering patterns that were highly congruent with those obtained from the hierarchical cluster analysis, suggesting strong agreement between the two methods (Figure 8 and Figure S12).

The prediction capacity of linear discriminant analysis (LDA) was used to validate the hierarchical cluster analysis (Table S6). The distribution of the genotypes within the clusters was verified and compared to identify the misclassified genotypes and to re-assigned them to suitable groups based on the confusion matrix of LDA. The confusion matrix indicated that genotypes were assigned to distinct clusters with 93% overall correctness (Table S6).

### 3.6. Cluster-Specific Trait Responses to Drought

**Figure 9:**
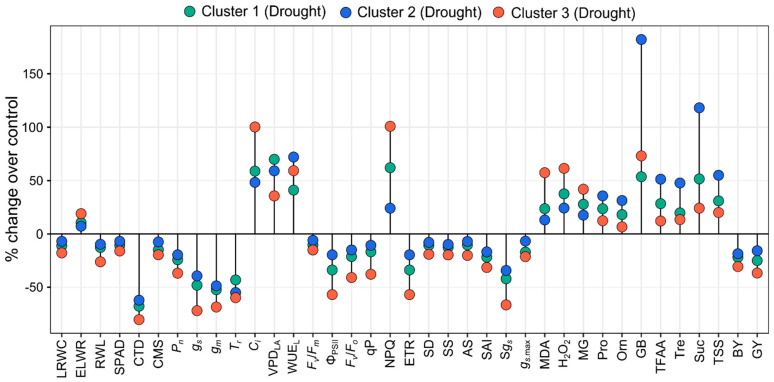
% change in studied traits due to drought over control of wheat genotypes under three clusters. Additional details are shown in Figure 3, Figure 4, Figure 5, Figure 6 and Figure 7.

Drought stress decreased the LRWC, RWL, SPAD, CTD, and CMS while increasing the ELWR, however, the magnitude was smaller in Cluster 2 genotypes than in Cluster 1 and 3 (Figure 9). Under both growing conditions, there was a gradual decrease in SPAD and CTD from heading to physiological maturity in the genotypes of all three clusters (Figure S13). The magnitude of the decrease in SPAD and CTD was the greatest in genotypes from Cluster 3, intermediate in genotypes from Cluster 1, and lowest in genotypes from Cluster 2 (Figure S13). *P_n_*, *g_s_*, *g_m_*, and *T_r_* declined in response to drought, with a lesser degree in Cluster 2 genotypes, while the lowest reduction in *T_r_* was recorded in Cluster 1 genotypes (Figure 9). Under drought conditions, Clusters 1, 2, and 3 genotypes showed the lowest increases in WUE_L_, *C_i_*, and VPD_LA_, respectively (Figure 9). Drought stress negatively affected all stomatal characteristics, with Cluster 2 genotypes exhibiting a lesser decline than Cluster 1 and 3 genotypes (Figure 9). Under drought stress, genotypes belonging to cluster 2 showed the lowest (9%) decrease in SD followed by genotypes of cluster 1 (12%) and 3 (16%) (Figure 9). A marked decrease in SS, AS, SAI, S*g_s_*, and g*_s_*_.max_ was recorded under drought, with the lowest decrease in the genotypes of cluster 2 and hereafter genotypes of clusters 1 and 3 (Figure 9). In response to drought, Cluster 2 genotypes exhibited minimal changes in *F*_v_/*F*_m_ (3%), Φ_PSII_ (20%), *F*_v_/*F*_o_ (15%), qP (11%), and ETR (20%) than the genotypes of Cluster 1 and 3, while Cluster 3 genotypes showed the highest increase (101%) in NPQ (Figure 9). During drought stress, a significantly lower increase in prooxidants contents viz. MDA (13%), H_2_O_2_ (24%), and MG (18%) were noted in the genotypes of cluster 2 compared to other clusters (Figure 9). A marked increase in the osmolyte content was recorded in response to drought, with a higher increase in the Cluster 2 genotypes than in genotypes of other clusters (Figure 9). In response to drought stress, the BY and GY decreased significantly in all clusters, albeit to varying degrees (Figure 9). Drought reduced BY by 22, 19, and 31% in clusters 1, 2, and 3, respectively, while grain yield declined by 25, 16, and 37% in clusters 1, 2, and 3, respectively (Figure 9).

Altogether, Cluster 2 genotypes (BARI Gom 26, BARI Gom 33, BD-631, BD-600, BD-9910, BD-9889, and BD-637) possess relatively greater potential in drought tolerance than the genotypes of Cluster 1 and 3, as evidenced by the minimal changes in physiological traits, gas exchange parameters, ChlF parameters, stomatal characteristics, lower ROS concentration, higher accumulation of osmolytes and greater yield stability under drought. On the other hand, Cluster 1 genotypes (BD-477, AS-10632, BD-666, BD-525, BD-466, and BAW-1147) were rated intermediately drought-tolerant, whereas the solitary genotype AS-10617 of Cluster 3 was deemed drought-susceptible because of its sensitive responses to drought.

### 3.7. Genotype Selection and Ranking Based on Drought Tolerance

**Figure 10:**
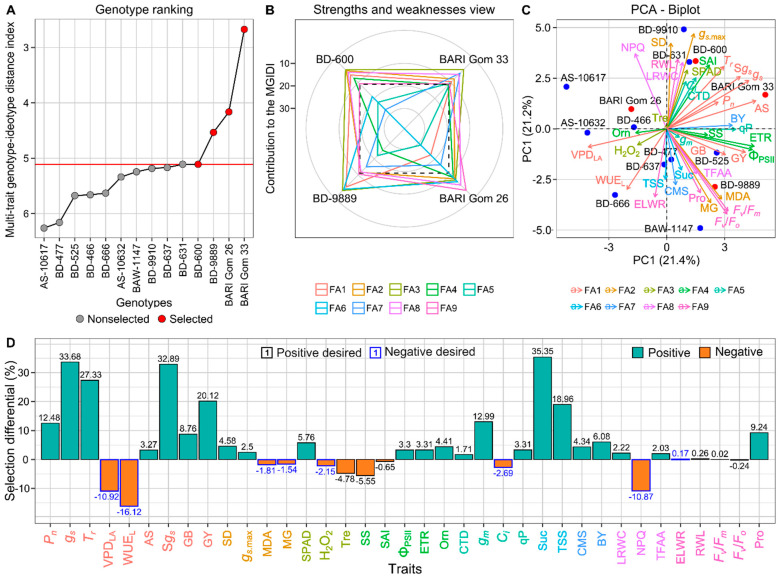
Genotype ranking based on MGIDI analysis (**A**). The selected genotypes are highlighted in red circle, red line denotes the cut point determined by the selection pressure. Selected genotype’s strengths and weaknesses are expressed as proportions within the individual factor in the calculated MGIDI (**B**). The closer a trait is to the outer edge, the more closely it aligns with the ideal genotype, The dashed line represents the expected value when all factors are equally influential. The PCA biplot showcasing the studied traits and their corresponding factors (**C**). The vector colors on the biplot indicate how traits are positioned relative to the corresponding factors in the strength and weaknesses view of the MGIDI analysis. The selection differentials (%) for the studied traits were obtained through the selection of drought-tolerant wheat genotypes (**D**). Additional details are shown in Figure 3, Figure 4, Figure 5, Figure 6 and Figure 7.

Multivariate analysis of variance (MANOVA) clearly revealed that studied traits are highly significantly different (*p* < 0.01) among genotypes, environment, and their interaction (Table S7). Except for Φ_PSII_ and ETR, the broad-sense heritability (*H*^2^) for all other traits was notably high (ranging from 54 to 96%), indicating their suitability for breeding improvement (Figure S14). Figure 10A shows the genotype ranking according to the multi-trait genotype-ideotype distance index (MGIDI). The genotype scoring a lower MGIDI is considered to be closest to the ideotype genotype and exhibited desirable values for all the studied traits. The MGIDI identified four drought-tolerant genotypes from this investigation: BARI Gom 33, BARI Gom 26, BD-9889, and BD-600 (Figure 10A). Interestingly, all these selected genotypes originated from Cluster 2, which was the highest performing group identified earlier in the hierarchical cluster analysis (Figure 8).

The MGIDI algorithm employed factor analysis to categorize 37 traits into 9 factors (FA) (Figure 10B–D). Factor analysis revealed distinct profiles for different FAs, providing insights into plant traits associated with growth and stress tolerance. FA1 highlights reduced water stress (lower VPD_LA_, WUE_L_) and enhanced growth potential (higher *P_n_*, *g_s_*, *T_r_*, AS, S*g_s_*, GB, and GY). Similarly, FA2 indicates lower oxidative stress (MDA and MG) and higher conductance (SD and *g_s.max_*). FA3 is linked to reduced oxidative stress (lower H_2_O_2_ and Tre) and higher pigments (SPAD). Interestingly, FA4 associated with lower stomatal area (SS and SAI) and improved photosynthetic efficiency (higher Φ_PSII_, ETR, and Orn). FA5 indicates lower *C_i_* and cooler canopy with improved photochemical efficiency (higher CTD, *g_m_*, and qP). FA6 highlights higher sugar content (higher Suc and TSS), while FA7 suggests membrane stability and biomass (higher CMS and BY). FA8 is linked to reduced non-photochemical quenching (lower NPQ) and better water retention (higher LRWC and TFAA), and FA9 indicates reduced energy conversion (lower *F_v_*/*F_o_*) and enhanced hydration state with higher photosynthetic performance (higher ELWR, RWL, *F_v_*/*F_m_*, and Pro). Loadings for these factors were presented in Table S8.

The contribution of individual factor to the MGIDI is presented in strengths and weaknesses view (Figure 10B) and PCA-biplot (Figure 10C). In this figure, the closer a trait is to the outer edge, the more closely it aligns with the ideal genotype, reflecting a lower proportion of the factor’s explanatory power. The dashed line represents the hypothetical value assuming all factors were equally influential. Analysis of genotype strengths and weaknesses revealed variations in FA contributions (Figure 10B). BARI Gom 33 showed the least influence from FA3, FA5, FA7, and FA8. Similarly, FA4, FA8, and FA9 played a minimum significant role in BARI Gom 26. For BD-9889, FA1, FA3, and FA6 were the least influential factors. Interestingly, BD-600 consistently exhibited lower contributions from FA3, FA4, and FA8.

The MGIDI offered expected selection differentials (SDs) for 32 out of 37 studied traits (Figure 10D). For traits with a target of lower values were desired (8 traits), the SDs ranged from −16.12% (WUE_L_) to 0.17% (ELWR). Conversely, for traits that should have higher desired values (29 traits), the SDs ranged from −5.55% (SS) to 35.35% (Suc). In this study, the MGIDI achieved high SDs, with a total of 247.70% for traits targeted for increase and −45.92% for traits targeted for decrease (Figure 10D). Traits related to photosynthetic efficiency (*P_n_*, SPAD, NPQ), biomass accumulation (BY, GY), leaf conductance (*g_s_*, *T_r_*, S*g_s_*, *g_m_*), water stress (VPD_LA_, WUE_L_), and osmoprotection (Suc, TSS, Pro, GB) displayed higher SDs in MGIDI rankings, indicating greater variability among genotypes for these traits (Figure 10D).

## 4. Discussion

### 4.1. Tissue Hydration and Cooling: Key Factors in Drought Tolerance

Leaf relative water content (LRWC) is a crucial indicator of drought resilience in wheat [81,82]. Higher LRWC was observed in drought-tolerant genotypes (Cluster 2) compared to the susceptible genotypes in Clusters 1 and 3. Excised leaf water retention (ELWR) increased while relative water loss (RWL) decreased under drought, suggesting leaf rolling as a water conservation mechanism [83,84]. In the present investigation, drought-tolerant genotypes showed minimal changes in ELWR and RWL, indicating efficient water management under drought. Flag leaf chlorophyll content decreased in response to drought in many crops, including wheat [82,85]. The drought-induced decline in the SPAD-chlorophyll index was less pronounced in Cluster 2 genotypes, indicating their capacity to maintain greenness and photosynthetic activity longer. While drought-tolerant wheat genotypes showed reduced chlorophyll loss compared to their susceptible counterparts [86], canopy temperature depression (CTD) and cell membrane stability (CMS) were key physiological markers of heat stress and cellular stability [6,7,82]. This study showed that drought decreased CTD and CMS in all genotypes, but with a lesser magnitude of reduction in Cluster 2 genotypes, demonstrating their capacity to maintain tissue cool and hydrated, and reduce membrane damage during drought. The limited changes in LRWC, ELWR, and RWL reinforce the ability of Cluster 2 genotypes to sustain optimal hydration and cooling conditions. Decreased LRWC, CTD, and CMS restricted biomass formation under drought, as evidenced by significant positive correlations of biological yield (BY) and grain yield (GY) with LRWC (*p* < 0.05 and *p* < 0.05), CTD (*p* < 0.05 and *p* < 0.05), and CMS (*p* < 0.05 and *p* < 0.01), respectively (Table 1). Hence, the relatively minor changes in water retention, lower chlorophyll decline, a cooler canopy, and more stable membrane system played key roles in the drought resistance of Cluster 2 genotypes.

### 4.2. Photosynthesis and Leaf Conductance: Crucial for Drought Tolerance

Results of the current study exhibited that, drought stress substantially declined *P_n_*, *g_s_*, *g_m_*, and *T_r_* in wheat (Figure 3). This was accompanied by an increase in intercellular CO_2_ concentration, VPD_LA_, and WUE_L_. Previous studies have reported similar findings [21,85,87]. The reduction in *P_n_*, in this study, was primarily attributed to decreased chlorophyll content (SPAD index) and restricted CO_2_ diffusion due to reduced stomatal aperture space (AS), *g_s_*, and *g_m_*. Drought-induced stomatal closure limited water loss and conserved water, leading to lower *g_s_* and *g_m_*. This, in turn, reduced chloroplast CO_2_ concentrations and carboxylation [88]. A significant correlation between *P_n_* and SPAD Chl index (*p* < 0.01) confirmed the importance of chlorophyll content in photosynthesis under drought conditions (Figure S10A).

Reduced *g_s_* in this study was mainly driven by drought-induced stomatal closure, limiting water loss through transpiration [85]. This was linked to decreases in AS, SAI, S*g_s_*, and maximum stomatal diffusive conductance (*g_s_*_.max_), collectively lowering *P_n_*. A solid positive correlation between *P_n_* and *g_s_* (*p* < 0.001) highlighted *g_s_* as a major driver of *P_n_* under drought (Figure S10B). Reduced *P_n_* and *g_s_* also limited biomass accumulation, as shown by significant positive correlations between BY and GY with *P_n_* (*p* < 0.05 and *p* < 0.01) and *g_s_* (*p* < 0.001 and *p* < 0.001) (Table 1). Drought significantly decreased *g_m_*, likely due to variability in Rubisco activity and leaf anatomy [89]. Both *g_s_* and *g_m_* contributed to the decline in *P_n_* [30,90], but a stronger correlation (r = 0.75, *p* < 0.001) between *P_n_* and *g_m_* suggested mesophyll carboxylation efficiency was more critical under drought (Figure S10C). The reduction in *T_r_*, driven by stomatal closure, further limited CO_2_ diffusion [91], reducing *P_n_*. Significant positive correlations between *P_n_* and traits like *T_r_*, AS, *g_s_*, and S*g_s_* supported these findings (Figure S10B,D,I,J). In the current study, *C_i_* remained high under drought conditions. This indicated non-stomatal limitations, such as decreased adenosine triphosphate (ATP) and Rubisco activity, were primarily responsible for the reduction in *P_n_* [43,87]. A significant negative association (*p* < 0.05) between *P_n_* and *C_i_* under drought suggested that the higher amount of CO_2_ in the stomatal cavity was not undergoing carboxylation due to enzyme inactivation (Figure S10E). VPD_LA_ increased under drought conditions, contributing to stomatal closure and leaf dehydration. Although VPD_LA_ increased, plants transpired more despite the lower diffusional conductance (*g_s_* and *g_m_*) [92]. A significant negative correlation (*p* < 0.05) between *P_n_* and VPD_LA_ was observed under both control and drought conditions (Figure S10F). WUE_L_ significantly increased under terminal drought stress due to a faster decrease in *g_s_* (44%) and *T_r_* (50%) compared to *P_n_* (24%). This was consistent with previous findings [43,93]. WUE_L_ was strongly but inversely related to *P_n_* under both control (*p* < 0.001) and drought (*p* < 0.05) conditions (Figure S10G), and a similar correlation between *P_n_* and WUE_L_ was also reported by Wang et al. [12] in wheat. Genotypes in Cluster 2 showed improved WUE_L_ under drought, attributed to lower reductions in *P_n_*, *g_s_*, AS, and *T_r_* compared to other clusters.

### 4.3. Photosystem Stability Is Vital for Drought Tolerance

In the present investigation, drought stress significantly decreased ChlF parameters, except for NPQ, which increased significantly. This aligns with previous findings of Terletskaya et al. [94], Barboričová et al. [95], Sherstneva et al. [1], and Ghaffar et al. [8]. Our study revealed that drought stress negatively impacts photosystem II (PSII) activity, leading to decreased *F_v_*/*F_m_*, *F_v_*/*F_o_*, Φ_PSII_, qP, and ETR, while increasing NPQ (Figure 4). This disruption is likely influenced by cyclic electron flux in chloroplast electron transport chains, resulting in lumen acidification and heat dissipation [96]. Consequently, the reduction in ETR under drought conditions may contribute to increased photoinhibition due to the accumulation of excess excitation energy around light-harvesting complex II (LHCII). Previous research has suggested that decreased ETR during drought is a result of downregulation of PSII and the Calvin cycle [21,97,98]. The observed decline in *F_v_*/*F_m_*, *F_v_*/*F_o_*, and ETR ultimately led to a reduction in net *P_n_* under drought, as evidenced by significant positive correlations (*r* = 0.38, *p* < 0.01; *r* = 0.33, *p* < 0.05; and *r* = 0.39, *p* < 0.01) (Figure S10K,M,P). Additionally, decreased LRWC was found to be associated with reduced *F_v_*/*F_m_* and ETR under drought [24].

Light-adapted ChlF parameters (Φ_PSII_ and qP) exhibited significant decreases under drought conditions, indicating reduced excitation energy capture and photochemical capacity in water-scarce environments [12]. The decline in Φ_PSII_ and qP was correlated with decreased *P_n_* (*r* = 0.31, *p* < 0.05 and *r* = 0.35, *p* < 0.05) (Figure S10L,N). Reduced PSII electron transport and activity may limit *P_n_* levels, ultimately impacting plant growth and grain yield [26]. Decreased *F_v_*/*F_m_* and Φ_PSII_ were significantly positively correlated with biomass accumulation (BY and GY) under drought conditions (*p* < 0.05), indicating their restrictive effects on plant growth and yield (Table 1). At low *C_i_*, a larger proportion of the electron flux is diverted from CO_2_ assimilation to O_2_ reduction, leading to inefficient electron distribution to carboxylation and oxygenation of Rubisco and consequently reduced biomass accumulation [99].

The qP parameter measures the light energy which is absorbed by PSII antenna pigments in relation to photochemical electron transfer [100]. A drop in qP values in our study suggests increased susceptibility of PSII to photoinhibition [101]. Therefore, maintaining a high qP value is crucial for plants to resist photoinhibition during drought. Findings revealed that NPQ increases in response to water deficit, often associated with excess light energy under drought stress caused by stomatal closure [1]. Previous studies have reported decreased qP and increased NPQ under stressful conditions [25,102]. The decline in photosynthetic efficiency is a secondary effect of this photoprotective reaction under drought stress [103] as reflected in the significant negative correlation between *P_n_* and NPQ (*r* = −0.61, *p* < 0.001) in the present study (Figure S10O).

Genotypes of cluster 2 showed the least changes in ChlF parameters due to drought stress, suggesting relatively less affected photosystems under water-limiting conditions (Figure 9). It has been reported that tolerant genotypes may exhibit minimal declines in *F_v_*/*F_m_* and ETR compared to susceptible genotypes [8,95].

### 4.4. Stomatal Acclimation Is Requisite for Drought Tolerance

In this study, drought stress significantly reduced SD and SS during the grain-filling period, consistent with findings that water deficits decrease SD and SS [30,104]. This reduction helps minimize water loss [105]. Franks et al. [28] also noted that lower SD reduces stomatal conductance, improving water use efficiency, aligning with this study’s results. Additionally, a strong negative correlation between SD and SS has been previously observed [30,91]. Our findings corroborate this relationship, with significant negative correlations observed under both control (*r* = −0.32, *p* < 0.05) and drought (*r* = −0.35, *p* < 0.05) conditions (Figure S11A). Decreased SD and SS appear to be drought adaptation strategies in crop plants, contributing to balanced stomatal conductance and carbon fixation. This is evident in the significant positive correlations between SD and *P_n_* (*r* = 0.36, *p* < 0.05) and *g_s_* (*r* = 0.34, *p* < 0.05) under drought in our study (Figures S10H and S11D). Cluster 2 genotypes showed substantially higher SD than clusters 1 and 3, with a lesser decline under drought (Figure 9), implying better stomatal regulation of the genotypes. Drought-tolerant wheat genotypes often have greater SD than drought-susceptible genotypes [106,107].

Stomatal aperture space (AS) directly influences the rate of gas exchange [104]. Under drought, AS was pivotal during the grain-filling stage, correlating with *P_n_* (*r* = 0.29, *p* < 0.05) and *g_s_* (*r* = 0.51, *p* < 0.001) (Figures S10I and S11F). Plants enhance WUE_L_ by decreasing AS and *T_r_* [28]. The observed decreases in SD and AS inhibited *T_r_* and *g_s_*, leading to increased WUE_L_ in the present investigation. Lower hydraulic conductivity and increased abscisic acid content reduced guard cell turgor pressure, restricting *g_s_* [108,109]. A high positive association between AS and *g_s_* (*r* = 0.51, *p* < 0.001) under water deficit conditions indicates that reduced AS is more relevant than reduced SS for decreasing *g_s_*, *P_n_*, and water loss, as the AS directly plays a balancing act between the inflow of CO_2_ and the outflow of water via transpiration.

The integrative stomatal area index (SAI), Specific stomatal conductance (S*g_s_*), and theoretical maximum stomatal diffusive conductance (*g_s._*_max_) decreased in all genotypes under drought. Stomatal size and density collectively influence SAI and *g_s._*_max_, with smaller SS and SD leading to reduced SAI and *g_s._*_max_ [30,110]. The significant positive correlations between SD and SAI (*r* = 0.33, *p* < 0.05) and *g_s._*_max_ (*r* = 0.78, *p* < 0.001) under drought support this (Figure S11B,C). Additionally, *g_s_* correlated significantly with SAI (*r* = 0.45, *p* < 0.01) and *g_s._*_max_ (*r* = 0.43, *p* < 0.01) (Figure S11G,I). However, SD and *g_s_* together determined S*g_s_*, which showed very strong correlations with *g_s_* under control (*r* = 0.97, *p* < 0.001) and water-limiting conditions (*r* = 0.99, *p* < 0.001) (Figure S11H). Our data also showed that reduced SD and AS restricted biomass accumulation during drought. Significant positive relationships between BY and GY with SD (*r* = 0.31, *p* < 0.05; *r* = 0.35, *p* < 0.05) and AS (*r* = 0.38, *p* < 0.01; *r* = 0.46, *p* < 0.01) corroborate this (Table 1). Overall, cluster 2 genotypes exhibited a comparably lower decrease in stomatal characteristics than clusters 1 and 3, suggesting their potential for stomatal acclimation to drier conditions and reduced water loss (Figure 9).

### 4.5. Tolerant Genotypes Demonstrate Reduced ROS Accumulation

Drought-stressed wheat plants experience elevated levels of reactive oxygen species (ROS), such as O_2_^•−^ and H_2_O_2_, which can lead to lipid peroxidation and the generation of malondialdehyde (MDA) in plant cells [32,33,111]. Additionally, toxic aldehydes like MG accumulate, disrupting cellular functions and impairing antioxidant defenses [35]. The observed significant increases in MDA, H_2_O_2_, and MG in drought-stressed wheat plants in this study is consistent with previous findings [112,113,114]. Cell membrane damage, often assessed by membrane leakage, is a common consequence of drought stress [115]. The observed decrease in CMS in this study is supported by the increased accumulation of MDA, H_2_O_2_, and MG under drought stress, which is more pronounced in genotypes of cluster 3 compared to genotypes of cluster 2. Our data revealed that elevated levels of ROS, specifically H_2_O_2_, negatively impact biomass accumulation (BY and GY) under drought conditions, as demonstrated by the significant negative correlations between H_2_O_2_ and BY (*r* = −0.31, *p* < 0.05) and GY (*r* = −0.36, *p* < 0.05) (Table 1).

### 4.6. Osmolytes and Sugars: Chief Contributors to Drought Tolerance

Drought stress led to increased accumulation of osmolytes like Pro, Orn, GB, and TFAA, which aid in osmotic adjustment and protect cellular components [37,43,116]. The observed increase in Pro and GB content under drought stress is likely crucial for tolerance due to their roles in osmotic adjustment, macromolecule protection, enzyme structure maintenance, methylglyoxal detoxification, membrane stability, and defense against oxidative damage [117,118]. Our findings suggest that the accumulation of these osmolytes positively contributes to biomass accumulation during drought, as evidenced by significant positive correlations between BY and GY and Pro (*r* = 0.23, *p* > 0.05; *r* = 0.48, *p* < 0.01) and GB (*r* = 0.30, *p* < 0.05; *r* = 0.36, *p* < 0.05) (Table 1). Sugar accumulation is a key indicator of drought tolerance in crops [86]. In this study, drought-stressed wheat genotypes showed increased levels of soluble sugars like trehalose (Tre), sucrose (Suc), and total soluble sugars (TSS). These sugars act as osmolytes, helping maintain cell turgor and plant water status during drought [119]. They also enhance drought tolerance by supporting antioxidant activities, maintaining redox balance, and improving photosynthetic efficiency [120,121,122].

Wheat genotypes with higher levels of metabolomic and compatible solutes show greater drought tolerance [14]. In this study, Cluster 2 genotypes exhibited elevated levels of Pro, Orn, GB, TFAA, Tre, Suc, and TSS, indicating superior drought resilience compared to Clusters 1 and 3. These results are consistent with previous research underscoring the role of these solutes in drought tolerance [14,37,43,123].

### 4.7. Effective Selection and Ranking of Genotypes Using MGIDI

Plant breeders aim to combine desirable traits into high-performing genotypes, but selecting ideotype genotypes is challenging with multiple traits [46]. Modern statistical tools like principal component analysis, factor analysis, and cluster analysis are useful for grouping traits and identifying genotypes. This study used a two-way hierarchical clustering, principal component analysis, and linear discriminant analysis approaches to categorize wheat genotypes but struggled to rank ideotype genotypes (Figure 10 and Figure S12). Recently, Olivoto and Lúcio [124] introduced the multi-trait genotype-ideotype distance index (MGIDI), which helps in selecting and ranking genotypes with multiple desired traits. This method is gaining popularity among breeders for crops such as strawberries [47,48], barley [46] wheat [125], black bean [126], and rice [127], due to its robustness and interpretability. The multi-trait genotype-ideotype distance index (MGIDI) calculates the Euclidean genotype-ideotype distance using factor analysis scores [124]. Genotypes with lower MGIDI values generally show better stability and stress tolerance. In this study, MGIDI ranked 14 wheat genotypes, identifying four top performers (BARI Gom 33, BARI Gom 26, BD-9889, and BD-600) and highlighting BD-631 as a promising genotype near the selection threshold (Figure 10A). A lower factor contribution for a genotype indicates its greater selectivity for the associated trait (Figure 10B,C). The MGIDI enables breeders to specify trait increases or decreases, aligning genotypes with breeding goals [48]. In our investigation, the MGIDI analysis met selection goals for 32 of 37 traits, achieving 88% and 90% for lower and higher trait values, respectively (Figure 10D). Traits related to photosynthesis (*P_n_*, SPAD, NPQ), biomass (BY, GY), conductance (*g_s_*, *T_r_*, S*g_s_*, *g_m_*), water stress (VPD_LA_, WUE_L_), and osmoprotection (Suc, TSS, Pro, GB) showed significant variability and high broad-sense heritability (*H*^2^), indicating their potential for breeding drought-tolerant wheat (Figure 10D).

## 5. Conclusions

Present study demonstrated that drought stress significantly impacted physiological processes, gas exchange, chlorophyll fluorescence, stomatal function, osmoprotection, and biomass accumulation during wheat grain filling. While the study was conducted in a controlled experimental setup with a relatively small sample size, the findings provide valuable insights into the physiological mechanisms underlying drought tolerance in wheat. Wheat genotypes exhibited varying responses to drought conditions, indicating distinct genetic potentials for drought tolerance. Comprehensive statistical analysis of flag leaf physiological and biochemical traits effectively identified seven drought-tolerant genotypes (BD-637, BD-9889, BARI Gom 26, BARI Gom 33, BD-600, BD-631, and BD-9910) from a larger set. MGIDI analysis further ranked wheat genotypes based on their drought tolerance, with BARI Gom 33, BARI Gom 26, BD-9889, and BD-600 emerging as top performers. Traits with high heritability and selection differentials identified through MGIDI (*P_n_*, SPAD, NPQ, *g_s_*, *T_r_*, S*g_s_*, *g_m_*, VPD_LA_, WUE_L_, Suc, TSS, Pro, GB, BY, and GY) are promising targets for breeding drought-tolerant wheat. These findings provide valuable insights for developing drought-resilient wheat cultivars.

## Figures and Tables

**Table 1:** Correlation coefficients of BY and GY with some selected physiological, gas exchange, stomatal, ChlF parameters, ROS, and metabolites of wheat at the rapid grain filling period under control and drought conditions.

Trait	Growing Condition	LRWC	CTD	CMS	*P_n_*	*g_s_*	SD	AS	*F_v_*/*F_m_*	Φ_PSII_	H_2_O_2_	Pro	GB
BY	Control	0.10	0.09	0.17	0.30 *	0.58 ***	0.26	0.25	0.09	0.16	−0.11	0.12	0.14
	Drought	0.33 *	0.34 *	0.30 *	0.32 *	0.63 ***	0.31 *	0.38 **	0.40 **	0.35 *	−0.31 *	0.23	0.30 *
GY	Control	0.21	0.19	0.26	0.43 **	0.50 ***	0.11	0.40 **	0.22	0.17	−0.26	0.40 **	0.22
	Drought	0.36 *	0.33 *	0.46 **	0.45 **	0.60 ***	0.35 *	0.46 **	0.38 **	0.32 *	−0.36 *	0.48 **	0.36 *

*, **, and *** denote statistically significant at the threshold of *p* < 0.05, *p* < 0.01, and *p* < 0.001, respectively. Additional details are presented in Figure 3, Figure 4, Figure 5, Figure 6 and Figure 7.

## Data Availability

The datasets used and/or analyzed during the current study are available from the corresponding author on reasonable request.

## Supplementary Materials

The following supporting information can be downloaded at: https://www.mdpi.com/article/10.3390/plants14010035/s1, Figure S1: Descriptive summary of the studied physiological, gas exchange, ChlF, and stomatal traits of 14 bread wheat genotypes under control and drought-treated conditions; Table S1: List and pedigree of 14 wheat genotypes used in the exploratory study; Figure S2: Descriptive summary of the studied reactive oxygen species, metabolomic solutes, and yield of 14 bread wheat genotypes under control and drought-treated conditions; Table S2: Physical and chemical properties of the experimental soil; Figure S3: Performance of physiological traits of 14 wheat genotypes grown in control and drought conditions measured at 14 days after anthesis (GS75); Table S3: Monthly average climatic data of the wheat-growing season and the averages of ten successive years in the experimental site (24.038° N, 90.397° E); Figure S4: Performance of gas exchange parameters of 14 wheat genotypes grown in control and drought conditions measured at 14 days after anthesis (GS75); Table S4: Variance components (%) of physiological, gas exchange, stomatal, ChlF parameters, and the osmolyte contents in the context of wheat genotypes × growing condition using the general linear model; Figure S5: Performance of chlorophyll *a* fluorescence parameters of 14 wheat genotypes grown in control and drought conditions measured at 14 days after anthesis (GS75); Table S5: Eigenvalues and eigenvectors were extracted for the first five principal components of a PCA conducted on relative trait values; Figure S6: Light micrographs (×100 magnification) illustrating the variability in stomatal number, size, and morphology among different wheat genotypes under control and drought stressed conditions; Table S6: LDA-based confusion matrix analysis of wheat genotypes clustered by HCA and PCA to assess classification accuracy; Figure S7: Performance of stomatal characteristics of 14 wheat genotypes grown in control and drought conditions measured at 14 days after anthesis (GS75); Table S7: Multivariate analysis of variance (MANOVA) of the effects of genotype, growing condition, and their interaction on the analyzed traits; Figure S8: Performance of reactive oxygen species and metabolomic solute accumulation in 14 wheat genotypes grown in control and drought conditions measured at 14 days after anthesis (GS75); Table S8: Factorial loadings after varimax rotation obtained in the factor analysis; Figure S9: Performance of biomass accumulation of 14 wheat genotypes grown in control and drought conditions measured at 14 days after anthesis (GS75); Figure S10: Scatterplots showing the association between *P_n_* and some selected traits; Figure S11: Scatterplots showing the association of SD and *g_s_* with other stomatal characteristics; Figure S12: PCA-Biplot of wheat genotypes and studied traits constructed from relative trait values; Figure S13: Cluster-wise changes in SPAD chlorophyll index (A) and canopy temperature depression [CTD] (B) of the wheat genotypes measured everyday after heading under control and drought stress; Figure S14: Broad-sense heritability (*H*^2^) of the studied traits across the growing environments.
