# Supplementary material for: Multi-Trait Index-Based Selection of Drought Tolerant Wheat: Physiological and Biochemical Profiling
Source: Plants (Basel). 2024 Dec 26;14(1):35. doi: 10.3390/plants14010035 (PMC11723105; doi:10.3390/plants14010035)
Supplement: Supplementary file 1 [file plants-14-00035-s001.zip › plants-3335467-supplementary.pdf]

Table S1. List and pedigree of 14 wheat genotypes used in the exploratory study

Sl. No.	Genotype	Source	Type	Pedigree/ Collected from	Drought Tolerance*
1	AS-10617	ACI Seed	Mutant	BARI Gom 25; mutagen: 1% EMS	Relatively Susceptible
2	AS-10632	ACI Seed	Mutant	BARI Gom 25; mutagen: 1% EMS	Relatively tolerant (RT)
3	BARI Gom 26	BWMRI	Variety	ICTAL 123/3/RAWAL 87//VEE/HD 2285	RT
4	BARI Gom 33	BWMRI	Variety	KACHU/SOLALA	RT
5	BAW-1147	BWMRI	Advanced line	WRS, Gazipur	RT
6	BD-466	PGRC, BARI	Accession	WRS, Gazipur	RT
7	BD-477	PGRC, BARI	Accession	WRS, Gazipur	RT
8	BD-525	PGRC, BARI	Accession	WRS, Gazipur	RT
9	BD-600	PGRC, BARI	Accession	WRS, Gazipur	RT
10	BD-631	PGRC, BARI	Accession	WRS, Gazipur	RT
11	BD-637	PGRC, BARI	Accession	WRS, Gazipur	RT
12	BD-666	PGRC, BARI	Accession	WRS, Gazipur	RT
13	BD-9889	PGRC, BARI	Accession	RARS, Ishurdi	RT
14	BD-9910	PGRC, BARI	Accession	RARS, Ishurdi	RT

ACI- Advanced Chemical Industries; BARI- Bangladesh Agricultural Research Institute; BWMRI- Bangladesh Wheat and Maize Research Institute; PGRC- Plant Genetic Resource Center; RARS- Regional Agricultural Research Station; WRS- Wheat Research Station.

*Categorized using spectral reflectance-based phenotyping (DOI: www.doi.org/10.7717/peerj.14421).

Table S2. Physical and chemical properties of the experimental soil.

Soil characteristics	Analytical values
Physical properties	
Particle size distribution	
% Sand	26
% Silt	50
% Clay	24
Textural class	Silt loam
Bulk density (g cm^{-3})	1.40
Particle density (g cm^{-3})	2.65
Field capacity (% by weight)	30.6
Chemical properties	
Soil pH (Soil: water = 1: 1.25)	5.8
Total N (%)	0.08
Organic C (%)	0.65
Available P (ppm)	18.0
Exchangeable K^{+} (meq 100 g^{-1})	0.46
Exchangeable Ca^{++} (meq 100 g^{-1})	8.75
Exchangeable Mg^{++} (meq 100 g^{-1})	2.46
Cation exchange capacity (meq 100 g^{-1})	20.1
Sulfur (ppm)	9.25
Zinc (ppm)	1.72
Boron (ppm)	0.74

Table S3. Monthly average climatic data of the wheat-growing season and the averages of ten successive years in the experimental site (24.038°N, 90.397°E).

**Month**	**Air Temperature (°C)**			**Soil Temp. (°C) (30 cm depth)**	**Humidity (%)**	**Total Rainfall (mm)**	**Daily mean evaporation (mm)**
	Max	Min	Mean				
Across two growing seasons							
November	29.4	17.7	23.6	23.9	88.3	11.7	2.4
December	25.8	14.2	20.0	19.8	88.3	4.9	1.6
January	24.9	11.1	18.0	16.9	86.7	0.0	1.7
February	28.2	15.1	21.7	19.2	84.9	7.5	2.7
March	31.9	19.3	25.6	23.2	84.0	19.6	3.9
The ten-year average							
November	29.0	18.9	23.9	24.9	84.3	15.9	2.2
December	25.0	13.8	19.4	20.9	86.6	8.3	1.5
January	22.9	11.4	17.2	18.2	87.2	3.2	1.6
February	27.7	15.6	21.6	20.5	83.0	11.0	2.8
March	31.7	20.9	26.3	23.7	80.2	50.2	3.8

Source: BSMRAU Weather Station, Department of Agricultural Engineering, BSMRAU.

Table S4. Variance components (%) of physiological, gas exchange, stomatal, ChlF parameters, and the osmolyte contents in the context of wheat genotypes × growing condition using the general linear model

Trait	Block	Genotype (G)	Growing condition (C)	G × C	Residual
df	2	13	1	13	55
LRWC	2.6	54.0***	40.8***	0.9	1.6
ELWR	1.5	28.7***	67.3***	0.8	1.6
RWL	1.3	41.5***	53.9***	1.1	2.2
SPAD	0.0	36.4***	61.7***	1.5***	0.4
CTD	0.0	0.5***	99.3***	0.2***	0.0
CMS	3.5	50.0***	42.0***	1.4	3.0
P_n	0.0	5.4***	93.9***	0.4	0.3
g_s	0.5	17.4***	80.1***	1.5***	0.4
g_m	0.8	1.6***	96.7***	0.6*	0.3
T_r	0.0	5.0***	93.9***	1.0***	0.1
C_i	0.1	2.8***	96.5***	0.4*	0.2
VPD	0.3	4.2***	94.5***	1.0***	0.0
WUE	1.4	7.4***	88.8***	1.9***	0.4
SD	0.4	15.7***	81.5***	1.2	1.3
SS	7.4	14.5***	75.7***	0.4	1.9
AS	2.5	24.6***	69.0***	1.1	2.7
SAI	2.7	5.9***	89.2***	1.0	1.2
Sg_s	0.7	24.2***	72.9***	1.5*	0.7
$g_{s.\max}$	0.2	9.0***	87.1***	2.5*	1.2
F_v/F_m	1.2	3.9**	91.1***	2.5*	1.2
Φ_{PSII}	0.2	2.5**	94.4***	1.2	1.7
F_v/F_o	1.2	3.7**	91.8***	1.8	1.5
qP	0.1	17.6**	73.3**	2.0	7.0
NPQ	11.0	21.2***	59.9***	3.2	4.7
ETR	0.2	2.5*	94.4***	1.2	1.7
MDA	0.0	14.9***	83.1***	1.5***	0.4
H_2O_2	0.0	11.9***	87.0***	1.0***	0.2
MG	0.0	9.6***	89.3***	0.8**	0.3
Pro	2.2	29.8***	66.4***	0.9	0.7
Orn	1.2	12.7***	84.4***	1.0	0.6
GB	0.1	5.1***	92.8***	1.8***	0.1
TFAA	1.6	12.1***	82.8***	1.4	2.1
Tre	1.2	16.9***	79.2***	1.1	1.5
Suc	0.0	17.0***	81.8***	1.0***	0.2
TSS	0.1	21.2***	77.7***	0.5	0.5
BY	0.3	9.0***	89.4***	0.5	0.7
GY	1.5	24.1***	72.0***	1.3	1.1

LRWC– leaf relative water content, ELWR– excised leaf water retention, RWL– relative water loss, SPAD– SPAD chlorophyll index, CTD– canopy temperature depression, CMS– cell membrane stability, P_n– net photosynthesis rate, g_s– stomatal conductance, g_m– apparent mesophyll conductance, T_r– transpiration rate, C_i– intercellular CO_2

concentration, VPD_{LA}– leaf-to-air vapor pressure deficit, WUE_L– leaf-level instantaneous water use efficiency, SD– stomatal density, SS– stomata size, AS– aperture space, SAI– stomatal area index, Sg_s– specific stomatal conductance, $g_{s.max}$– maximum stomatal diffusive conductance, F_v/F_m– maximal efficiency of PSII, Φ_{PSII}– actual/effective efficiency of PSII, F_v/F_o– maximum energy conversion potential of PSII, qP– photochemical quenching, NPQ– non-photochemical quenching, ETR– electron transport rate, MDA– malondialdehyde, H_2O_2– hydrogen peroxide, MG– methylglyoxal, Pro– proline, Orn– ornithine, GB– glycine betaine, TFAA– total free amino acids, Tre– trehalose, Suc– sucrose, TSS– total soluble sugars, BY– biological yield, and GY– grain yield.
*, **, and *** denote statistically significant at $p < 0.05$, 0.01, and 0.001, respectively.

Table S5. Eigenvalues and eigenvectors were extracted for the first five principal components of a PCA conducted on relative trait values, including physiological, gas exchange, chlorophyll fluorescence, stomatal, reactive oxygen species, and osmolyte traits.

**Variable**	**Principal components (PC)**				
	PC1	PC2	PC3	PC4	PC5
Extracted Eigenvalues	18.696	3.959	3.313	2.368	2.051
Explained variance (%)	50.5	10.7	8.9	6.4	5.5
Cumulative variance (%)	50.5	61.2	70.1	76.6	82.1
Traits	*Latent vectors*				
LRWC	0.801	-0.191	0.353	0.237	0.177
ELWR	-0.817	-0.195	0.147	0.110	-0.200
RWL	0.597	0.287	-0.089	-0.095	0.008
SPAD	0.678	-0.224	0.419	0.155	0.175
CTD	0.631	-0.240	0.045	-0.514	0.101
CMS	0.696	-0.444	0.261	0.271	0.167
P_n	0.531	0.305	0.053	0.296	-0.578
g_s	0.820	0.355	-0.239	0.071	0.064
g_m	0.697	0.249	0.342	-0.176	-0.325
T_r	-0.023	0.863	0.244	0.005	0.236
C_i	-0.791	-0.290	-0.292	0.123	0.080
VPD_{LA}	0.254	-0.143	0.399	-0.554	-0.244
WUE_L	0.242	-0.744	-0.210	0.138	-0.461
SD	0.838	0.329	0.222	0.121	0.058
SS	0.853	0.130	0.289	-0.214	0.076
AS	0.879	0.126	0.053	-0.270	-0.066
SAI	0.860	0.248	0.277	-0.063	0.085
Sg_s	0.787	0.365	-0.330	0.070	0.028
$g_{s.max}$	0.497	-0.252	0.404	0.533	-0.277
F_v/F_m	0.728	0.277	-0.314	-0.023	0.156
Φ_{PSII}	0.942	-0.052	-0.031	0.187	-0.213
F_v/F_o	0.688	0.218	-0.351	-0.015	0.186
qP	0.833	0.133	0.284	0.078	-0.301
NPQ	-0.381	0.259	0.542	-0.064	0.402
ETR	0.942	-0.052	-0.031	0.187	-0.213
MDA	-0.820	0.059	0.138	0.469	0.091
H_2O_2	-0.777	0.154	0.022	0.343	0.319
MG	-0.753	0.237	0.156	0.528	0.055
Pro	0.808	-0.207	0.166	0.175	0.218
Orn	0.772	-0.321	0.162	0.107	0.300
GB	0.570	-0.386	-0.491	-0.066	0.216
TFAA	0.486	-0.439	-0.646	-0.074	0.138
Tre	0.821	-0.148	-0.155	0.307	0.316
Suc	0.714	-0.250	0.025	-0.012	0.430
TSS	0.785	-0.391	0.159	0.166	0.001
BY	0.561	0.491	-0.500	0.100	-0.154
GY	0.524	0.389	-0.501	0.397	-0.097

Additional details are shown in Table S4.

Table S6. LDA-based confusion matrix analysis of wheat genotypes clustered by HCA and PCA to assess classification accuracy. Rows being observed category and columns predicted category.

Predicted	Actual			Total no. observed
	Cluster 1	Cluster 2	Cluster 3	
Cluster 1	6	1	0	7
Cluster 2	0	6	0	6
Cluster 3	0	0	1	1
Total N	6	7	1	14
N correct	6	6	1	13
% correctness	100	86	100	93

Table S7. Multivariate analysis of variance (MANOVA) of the effects of genotype, growing condition, and their interaction on the analyzed traits

Effect	Value	Pillai's trace	Hypothesis DF	Error DF	P value
Intercept	186804.9	1.000	37	20	4.34×10^{-49}
Genotype (G)	4.776	11.007	481	416	1.58×10^{-54}
Growing condition (C)	257.787	0.998	37	20	1.64×10^{-20}
G × C	1.528	8.302	481	416	4.57×10^{-06}

Table S8. Factorial loadings after varimax rotation obtained in the factor analysis

Trait	FA1	FA2	FA3	FA4	FA5	FA6	FA7	FA8	FA9
P_n	**–0.64**	–0.38	0.11	0.14	–0.51	–0.30	0.06	0.17	0.19
g_s	**–0.95**	–0.02	0.05	0.11	0.08	0.04	–0.24	–0.02	–0.03
T_r	**–0.92**	–0.16	–0.03	–0.11	–0.16	–0.17	0.10	–0.05	–0.17
VPD_{LA}	**–0.76**	0.34	–0.08	0.02	0.20	–0.07	–0.37	–0.04	–0.13
WUE_L	**–0.78**	0.06	–0.13	–0.31	0.08	–0.09	–0.01	–0.08	–0.45
AS	**–0.68**	0.18	–0.31	0.37	–0.16	0.42	0.02	–0.11	0.21
Sg_s	**–0.97**	0.01	0.06	0.09	0.09	0.06	–0.06	–0.04	0.04
GB	**–0.49**	0.42	0.22	0.27	–0.12	–0.17	0.48	0.01	0.41
GY	**–0.56**	0.17	–0.24	–0.05	–0.27	–0.12	–0.46	–0.04	0.52
SD	–0.30	**–0.77**	–0.31	0.03	–0.04	0.04	–0.03	–0.44	0.02
$g_{s.max}$	–0.50	**–0.56**	0.15	0.39	–0.05	0.18	0.13	–0.31	–0.23
MDA	0.10	**–0.93**	–0.10	–0.13	–0.04	0.23	0.03	–0.03	–0.10
MG	0.08	**–0.93**	–0.10	–0.22	–0.01	0.21	0.06	–0.01	–0.12
SPAD	–0.35	–0.38	**–0.68**	0.03	–0.30	–0.12	0.26	–0.24	–0.05
H_2O_2	0.00	–0.08	**–0.89**	0.18	–0.04	0.22	–0.30	–0.13	0.05
Tre	–0.06	0.14	**0.95**	0.06	0.03	0.03	–0.09	–0.08	–0.20
SS	0.08	0.36	0.13	**0.83**	0.08	0.21	0.01	0.05	–0.24
SAI	–0.21	–0.27	–0.09	**0.82**	0.05	0.23	–0.01	–0.30	–0.21
Φ_{PSII}	–0.20	0.43	–0.15	**0.59**	–0.48	0.13	–0.34	–0.09	0.07
ETR	–0.20	0.43	–0.15	**0.58**	–0.49	0.13	–0.34	–0.09	0.07
Orn	–0.11	–0.07	0.09	**–0.92**	–0.20	0.03	–0.05	–0.08	0.18
CTD	–0.37	0.10	0.11	–0.08	**–0.51**	0.50	0.23	–0.32	–0.26
g_m	0.01	–0.25	–0.05	–0.10	**–0.92**	–0.16	–0.04	0.14	0.02
C_i	0.48	0.09	–0.14	–0.21	**–0.79**	0.02	–0.06	0.10	0.12
qP	–0.11	0.15	–0.33	0.49	**–0.53**	0.35	–0.42	–0.01	0.00
Suc	–0.21	0.30	0.10	–0.21	–0.11	**–0.85**	0.09	0.07	0.08
TSS	0.00	0.27	–0.01	–0.15	–0.01	**–0.94**	0.03	–0.01	–0.06
CMS	0.42	0.16	–0.44	0.08	–0.25	–0.20	**–0.47**	–0.16	0.35
BY	–0.43	0.05	0.06	0.05	–0.04	0.10	**–0.87**	–0.01	0.05
LRWC	–0.12	–0.15	–0.37	0.18	0.13	0.01	–0.20	**–0.75**	–0.30
NPQ	0.05	0.25	–0.07	0.12	–0.14	–0.41	–0.47	**0.64**	0.20
TFAA	–0.09	0.22	–0.30	–0.02	–0.41	0.36	0.21	**0.50**	0.45
ELWR	–0.22	–0.03	0.20	0.29	0.03	–0.03	0.03	–0.12	**–0.88**
RWL	–0.23	–0.02	0.15	0.31	0.03	–0.04	0.02	–0.15	**–0.87**
F_v/F_m	–0.01	0.49	0.08	0.47	–0.02	–0.11	–0.37	0.32	**0.53**
F_v/F_o	–0.02	0.51	–0.06	0.42	–0.01	–0.07	–0.29	0.35	**0.58**
Pro	–0.32	0.48	0.24	–0.25	0.06	–0.29	0.05	0.07	**0.62**

Additional details are shown in Table S4.

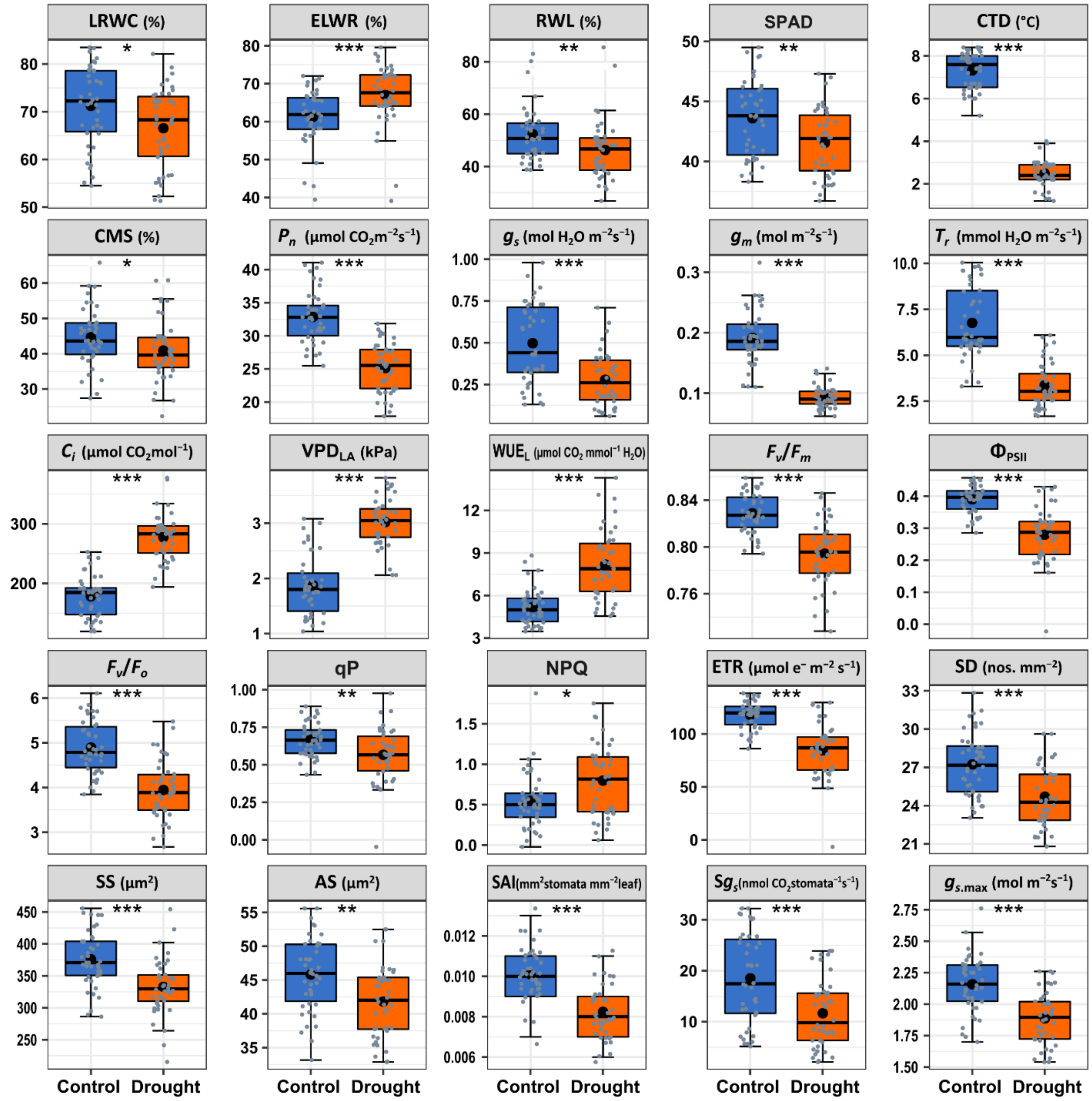

Figure S1. Descriptive summary of the studied physiological, gas exchange, ChlF, and stomatal traits of 14 bread wheat genotypes under control and drought-treated conditions. *, **, and *** denote statistically significant at $p < 0.05$, 0.01, and 0.001, respectively. Within the box, the horizontal thicker line and circle indicate the median and mean, respectively. The box's bottom and upper boundaries, as well as the lower and upper whiskers, correspond to Q1 (first quartile/25th percentile), Q3 (third quartile/75th percentile), (Q1 – 1.5IQR), and (Q3 + 1.5IQR), respectively. IQR stands for interquartile range. The distribution of wheat genotypes (3 replicates) is shown by the slate color dots on the boxes. Additional details are shown in Table S3.

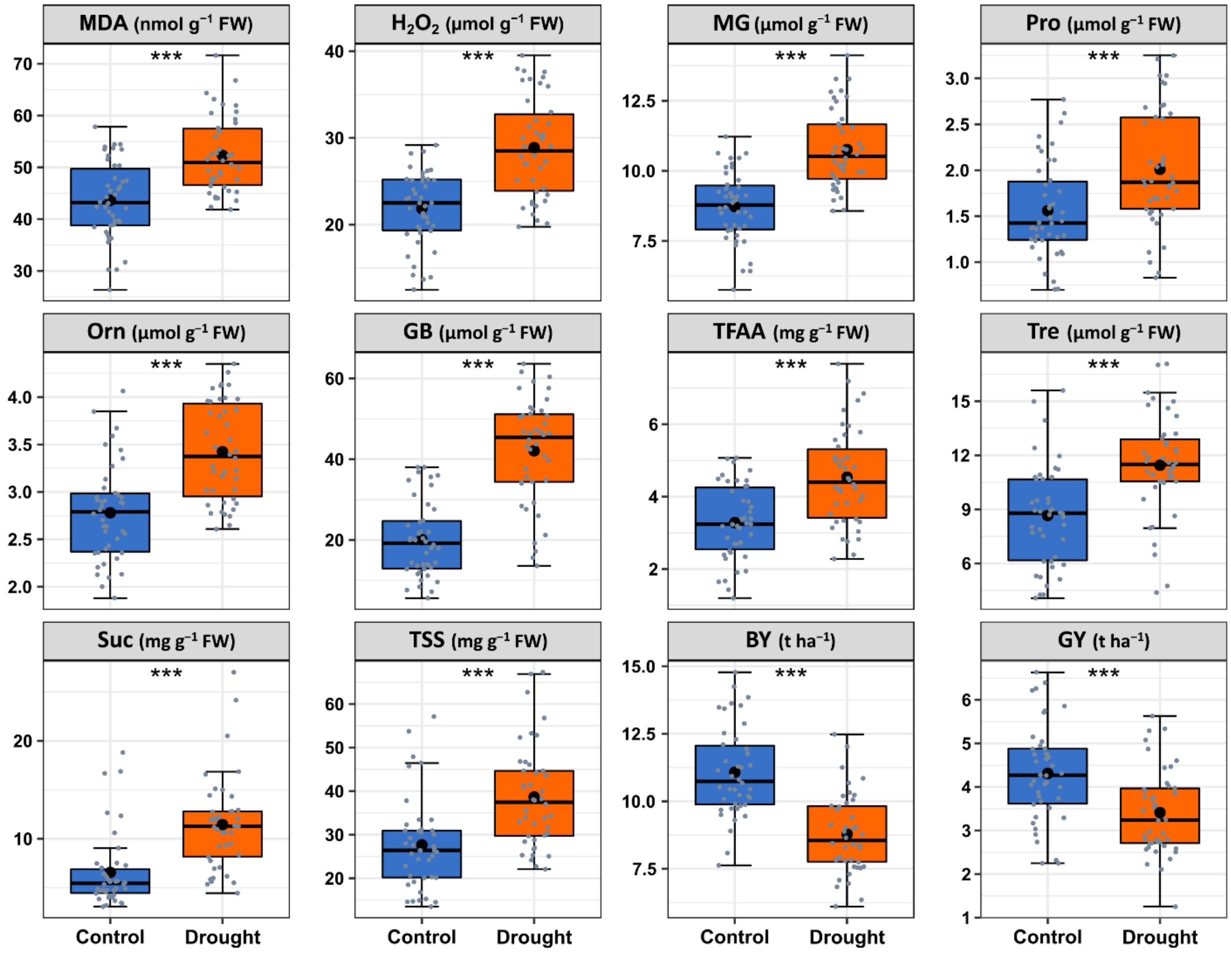

Figure S2. Descriptive summary of the studied reactive oxygen species, metabolomic solutes, and yield of 14 bread wheat genotypes under control and drought-treated conditions. *** denote statistically significant at $p < 0.001$. Within the box, the horizontal thicker line and circle indicate the median and mean, respectively. The box's bottom and upper boundaries, as well as the lower and upper whiskers, correspond to Q1 (first quartile/25th percentile), Q3 (third quartile/75th percentile), (Q1 – 1.5IQR), and (Q3 + 1.5IQR), respectively. IQR stands for interquartile range. The distribution of wheat genotypes (3 replicates) is shown by the slate color dots on the boxes. Additional details are shown in Table S3.

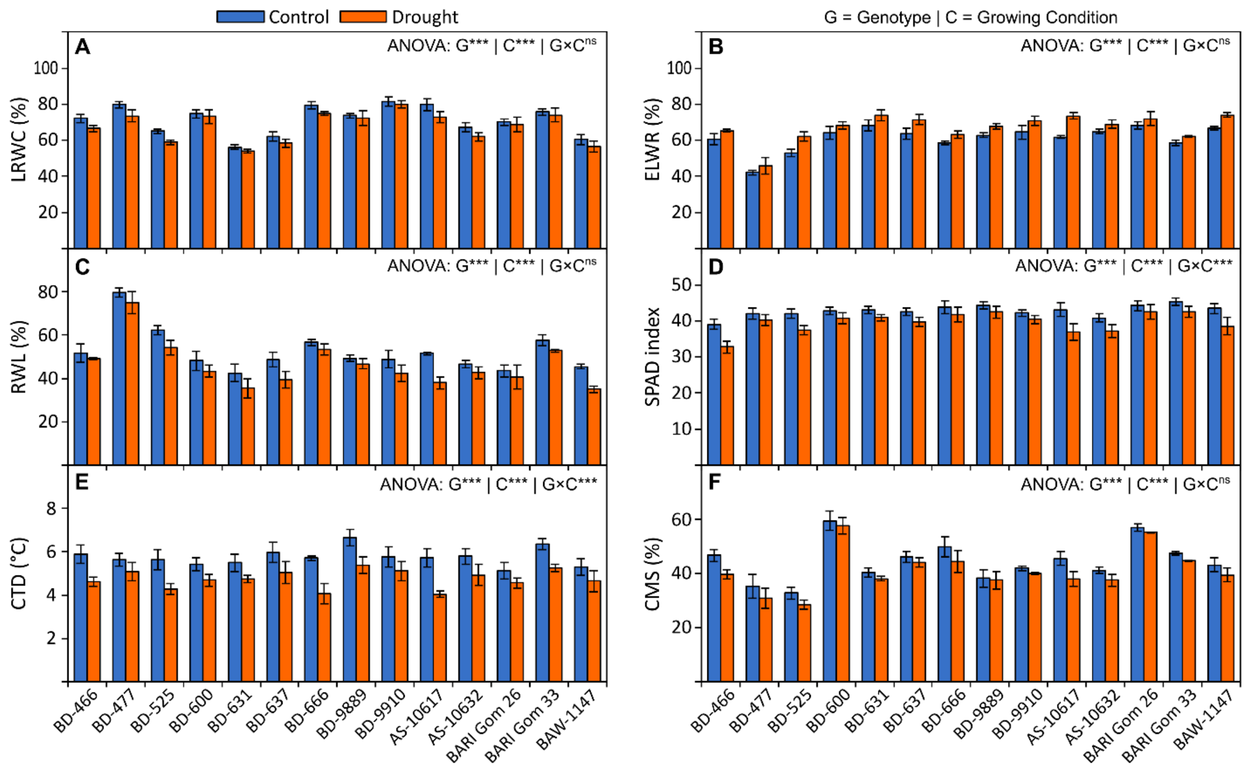

Figure S3. Performance of physiological traits of 14 wheat genotypes grown in control and drought conditions measured at 14 days after anthesis (GS75)- **(A)** leaf relative water content (LRWC), **(B)** excised leaf water retention (ELWR), **(C)** relative water loss (RWL), **(D)** chlorophyll index (SPAD), **(E)** canopy temperature depression (CTD), and **(F)** cell membrane stability (CMS). ^{ns} and *** denote non-significant and significant at a threshold of $p < 0.05$ and 0.001, respectively.

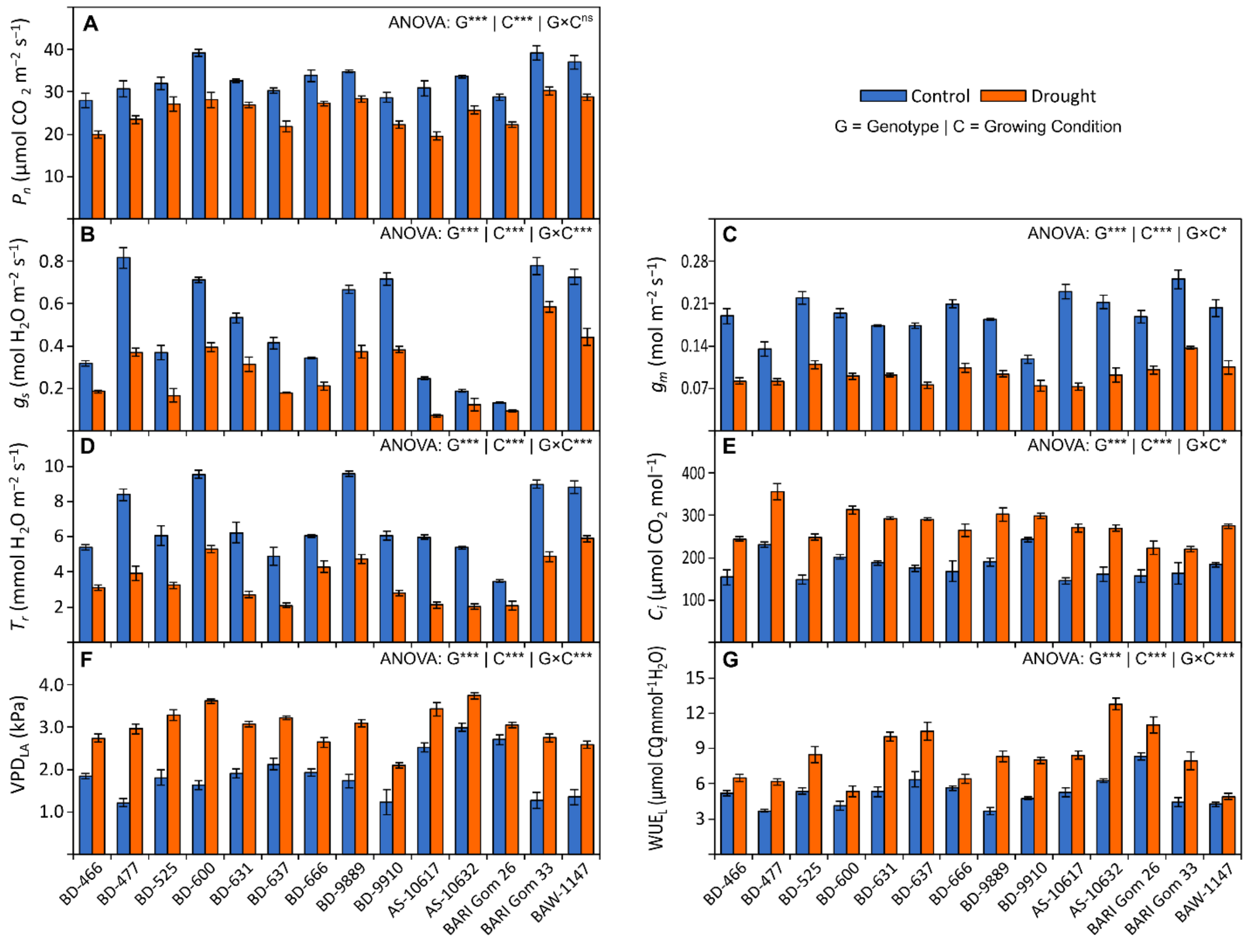

Figure S4. Performance of gas exchange parameters of 14 wheat genotypes grown in control and drought conditions measured at 14 days after anthesis (GS75)- **(A)** Net photosynthesis rate (P_n), **(B)** stomatal conductance (g_s), **(C)** apparent mesophyll conductance (g_m), **(D)** transpiration rate (T_r), **(E)** intercellular CO_2 concentration (C_i), **(F)** leaf-to-air vapor pressure deficit (VPD_{LA}), and **(G)** leaf-level instantaneous water use efficiency (WUE_L). ^{ns}, *, and *** denote non-significant and significant at $p < 0.05$, and 0.001, respectively.

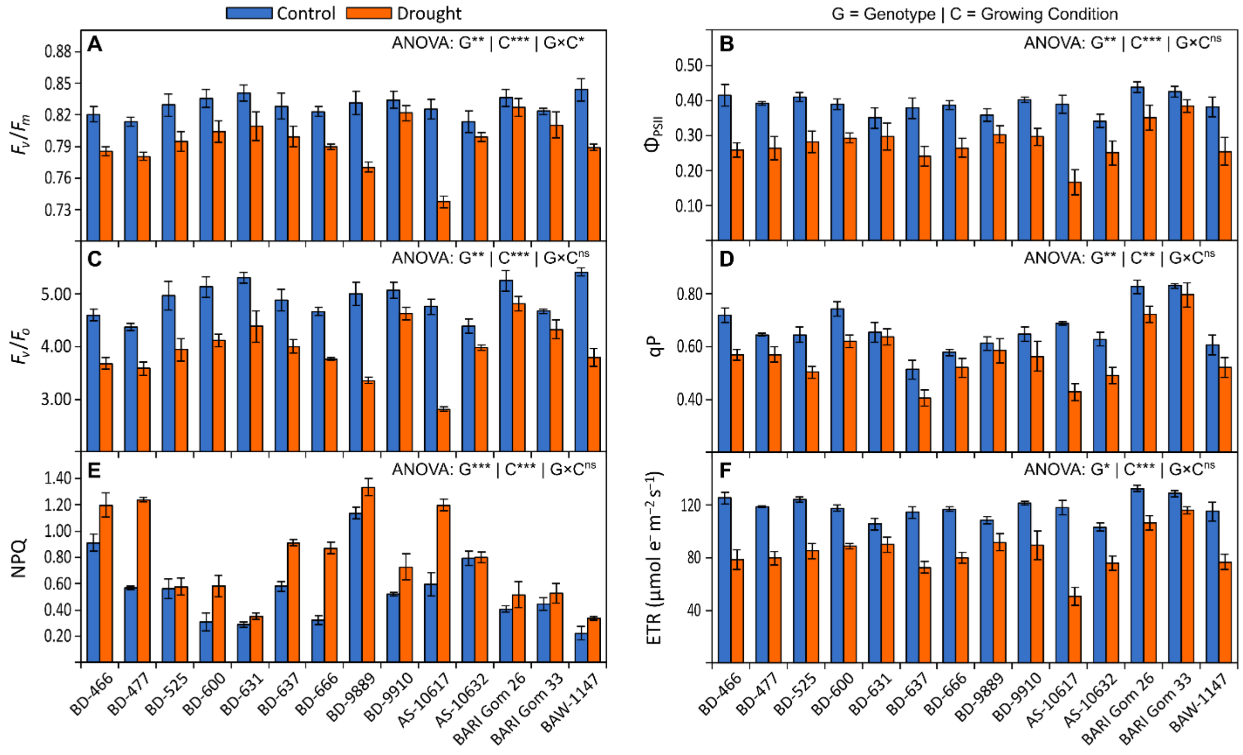

Figure S5. Performance of chlorophyll *a* fluorescence parameters of 14 wheat genotypes grown in control and drought conditions measured at 14 days after anthesis (GS75)- **(A)** maximal quantum efficiency of PSII (F_v/F_m), **(B)** quantum yield of PSII (Φ_{PSII}), **(C)** maximum energy conversion potential of PSII (F_v/F_o), **(D)** photochemical quenching (qP), **(E)** non-photochemical quenching (NPQ), and **(F)** electron transport rate (ETR). ^{ns}, *, **, and *** denote non-significant, significant at p < 0.05, 0.01, and 0.001, respectively.

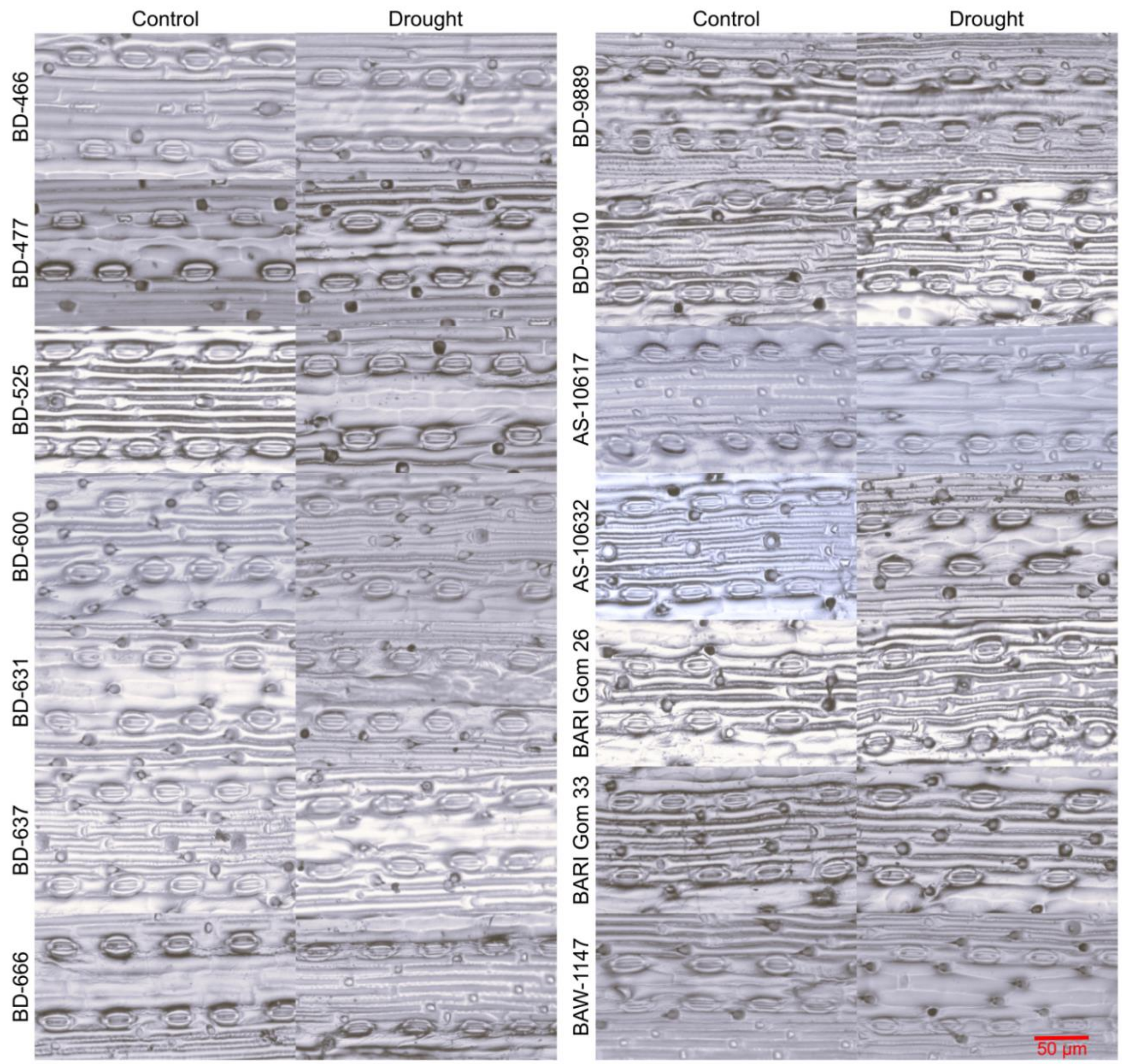

Figure S6. Light micrographs (×100 magnification) illustrating the variability in stomatal number, size, and morphology among different wheat genotypes under control and drought stressed conditions.

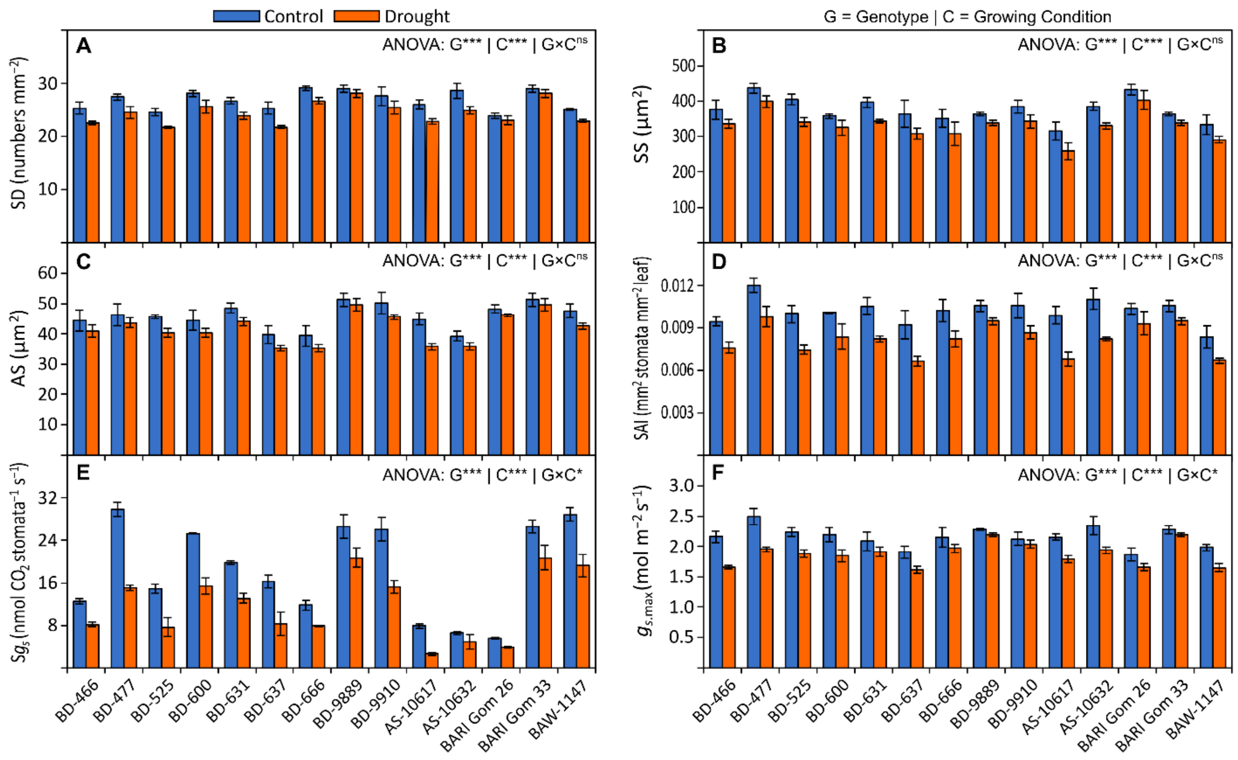

Figure S7. Performance of stomatal characteristics of 14 wheat genotypes grown in control and drought conditions measured at 14 days after anthesis (GS75)- **(A)** stomatal density (SD), **(B)** stomatal size (SS), **(C)** aperture space (AS), **(D)** stomatal area index (SAI), **(E)** specific stomatal conductance (Sg_s), and **(F)** maximum stomatal diffusive conductance ($g_{s.max}$). ^{ns}, *, and *** denote non-significant, significant at $p < 0.05$ and 0.001, respectively.

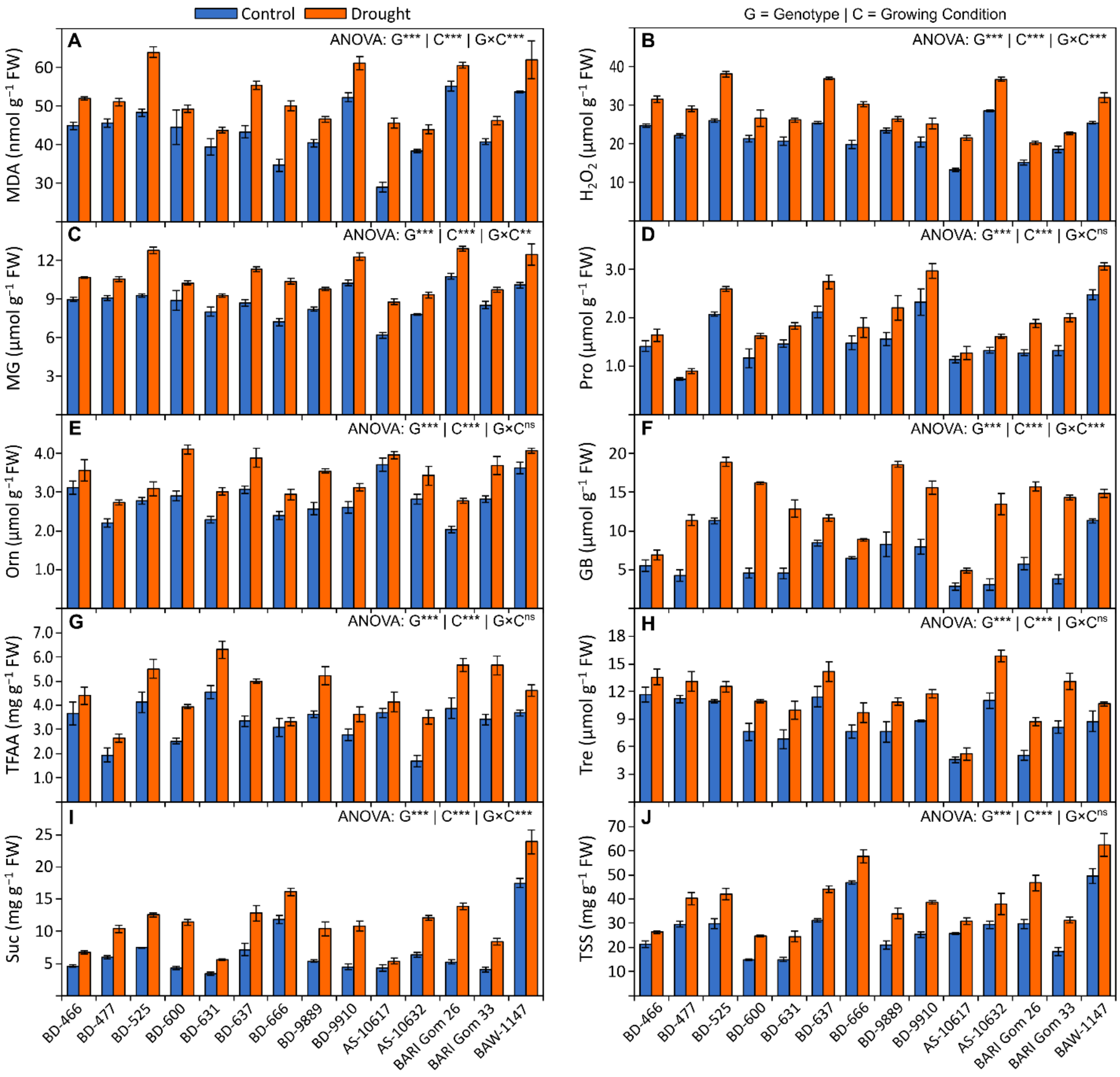

Figure S8. Performance of reactive oxygen species and metabolomic solute accumulation in 14 wheat genotypes grown in control and drought conditions measured at 14 days after anthesis (GS75)- **(A)** malondialdehyde (MDA), **(B)** hydrogen peroxide (H_2O_2), **(C)** methylglyoxal (MG), **(D)** proline (Pro), **(E)** ornithine (Orn), **(F)** glycine betaine (GB), **(G)** total free amino acid (TFAA), **(H)** trehalose (Tre), **(I)** sucrose (Suc), and **(J)** total soluble sugar (TSS). ^{ns}, **, and *** denote non-significant, significant at p < 0.01, and 0.001, respectively.

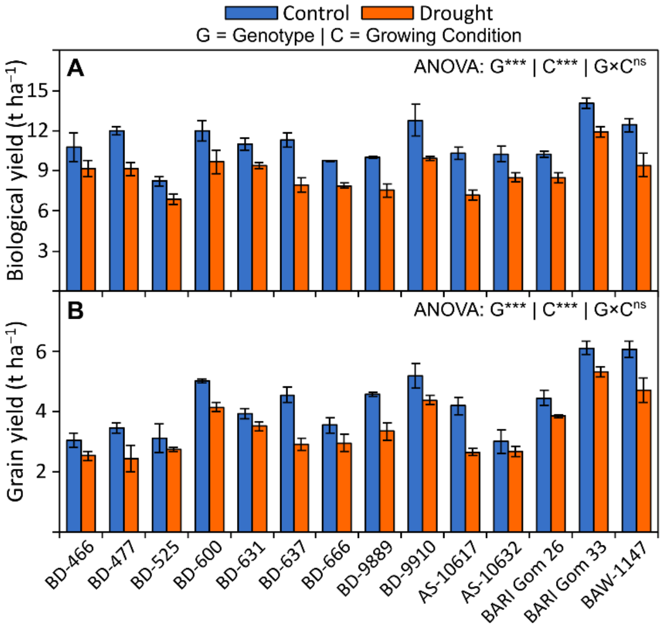

Figure S9. Performance of biomass accumulation of 14 wheat genotypes grown in control and drought conditions measured at 14 days after anthesis (GS75)- **(A)** biological yield (BY), and **(B)** grain yield (GY). ^{ns} and *** denote non-significant and significant at $p < 0.001$, respectively.

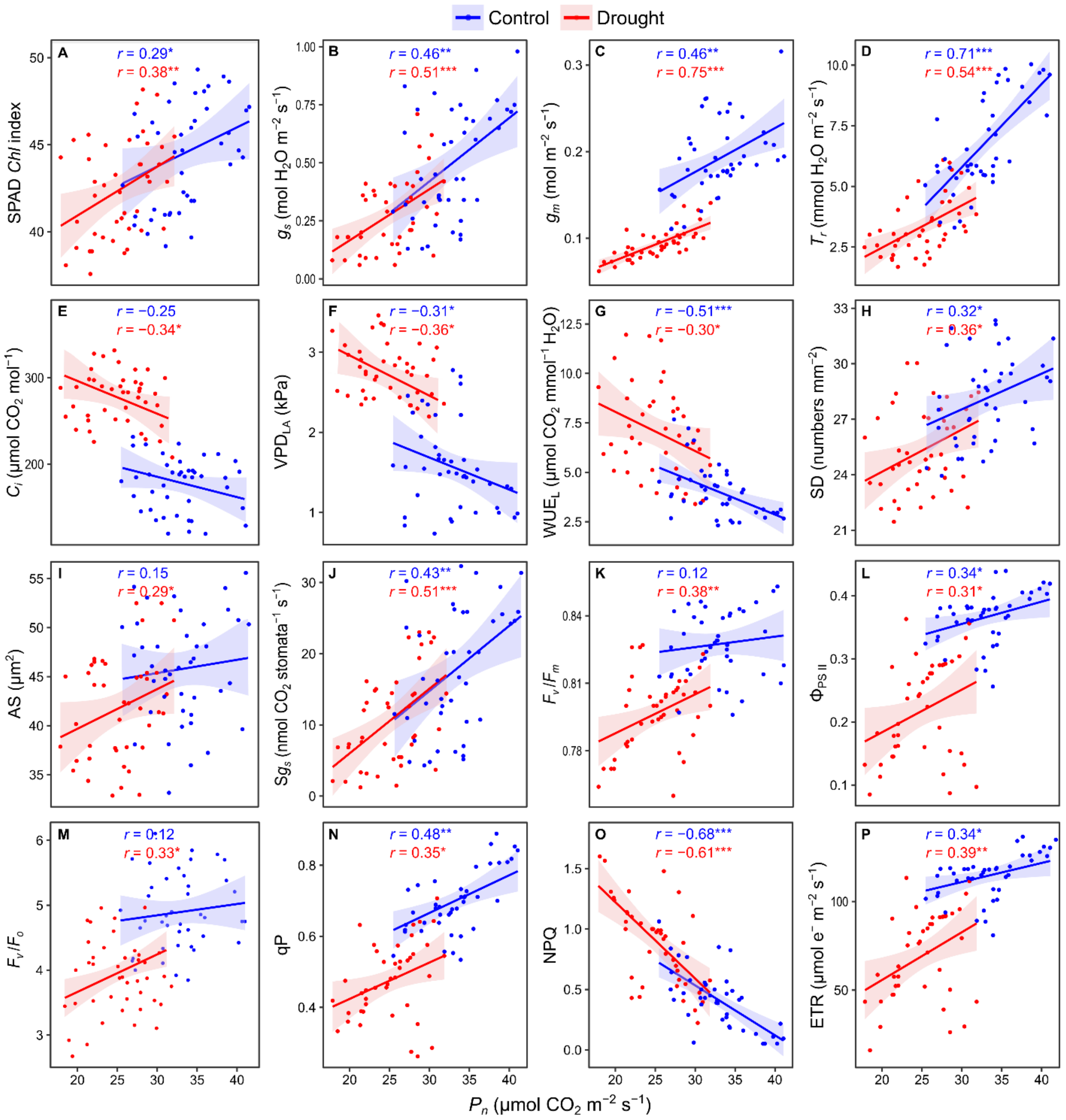

Figure S10. Scatterplots showing the association between P_n and some selected traits: SPAD *Chl* index **(A)**, gas exchange parameters **(B−G)**, stomatal characteristics **(H−J)**, and chlorophyll fluorescence parameters **(K−P)** under control (blue) and drought (red) conditions. *, **, and *** denote statistically significant at $p < 0.05$, 0.01, and 0.001, respectively. Additional details are shown in Figure 2−5.

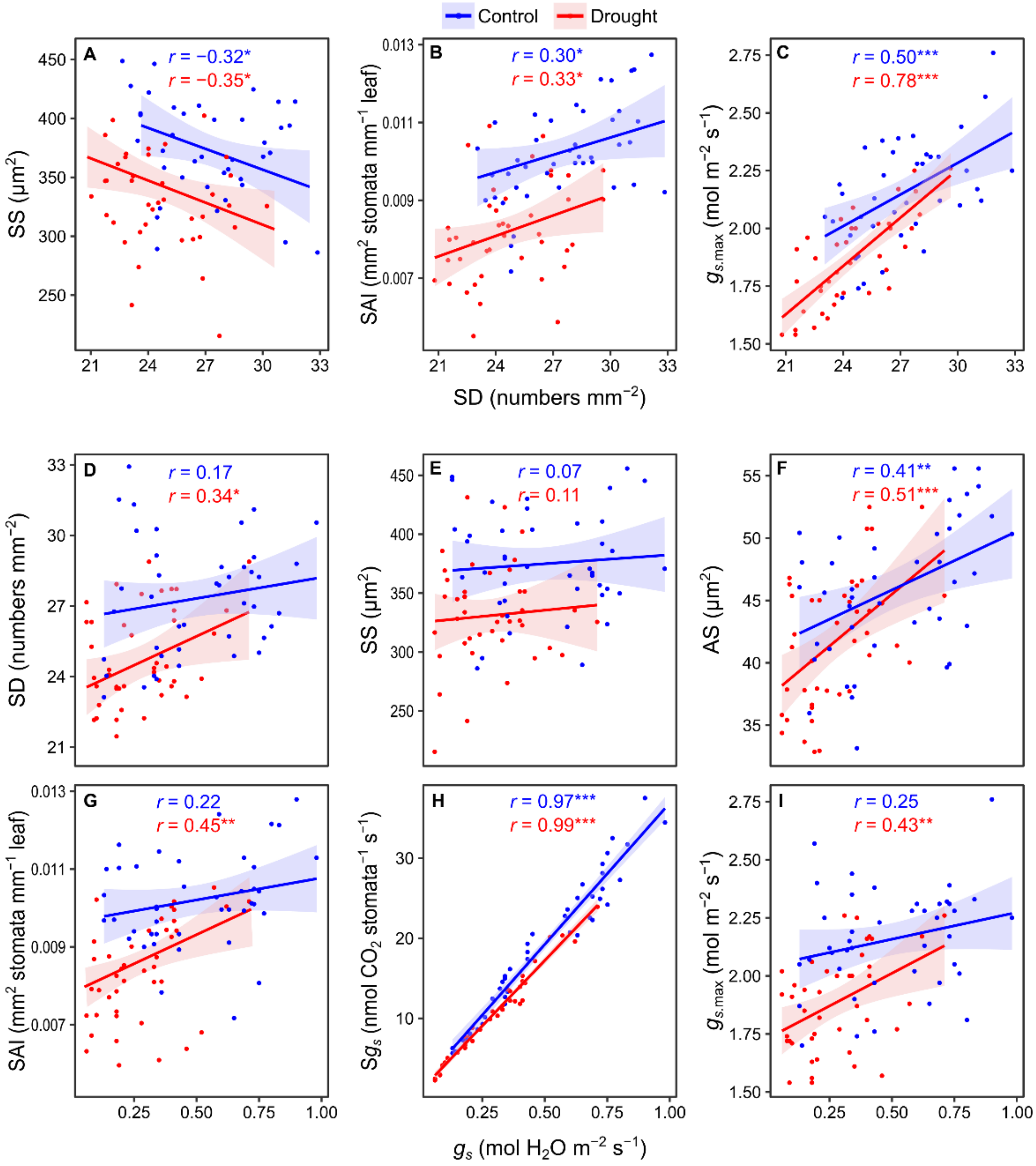

Figure S11. Scatterplots showing the association of SD with SS (**A**), SAI (**B**), $g_{s.\max}$ (**C**), g_s with SD (**D**), SS (**E**), AS (**F**), SAI (**G**), Sg_s (**H**) and $g_{s.\max}$ (**I**) under control (blue) and drought (red) conditions. *, **, and *** denote statistically significant at $p < 0.05$, 0.01, and 0.001, respectively. Additional details are shown in Figures 3 and 5.

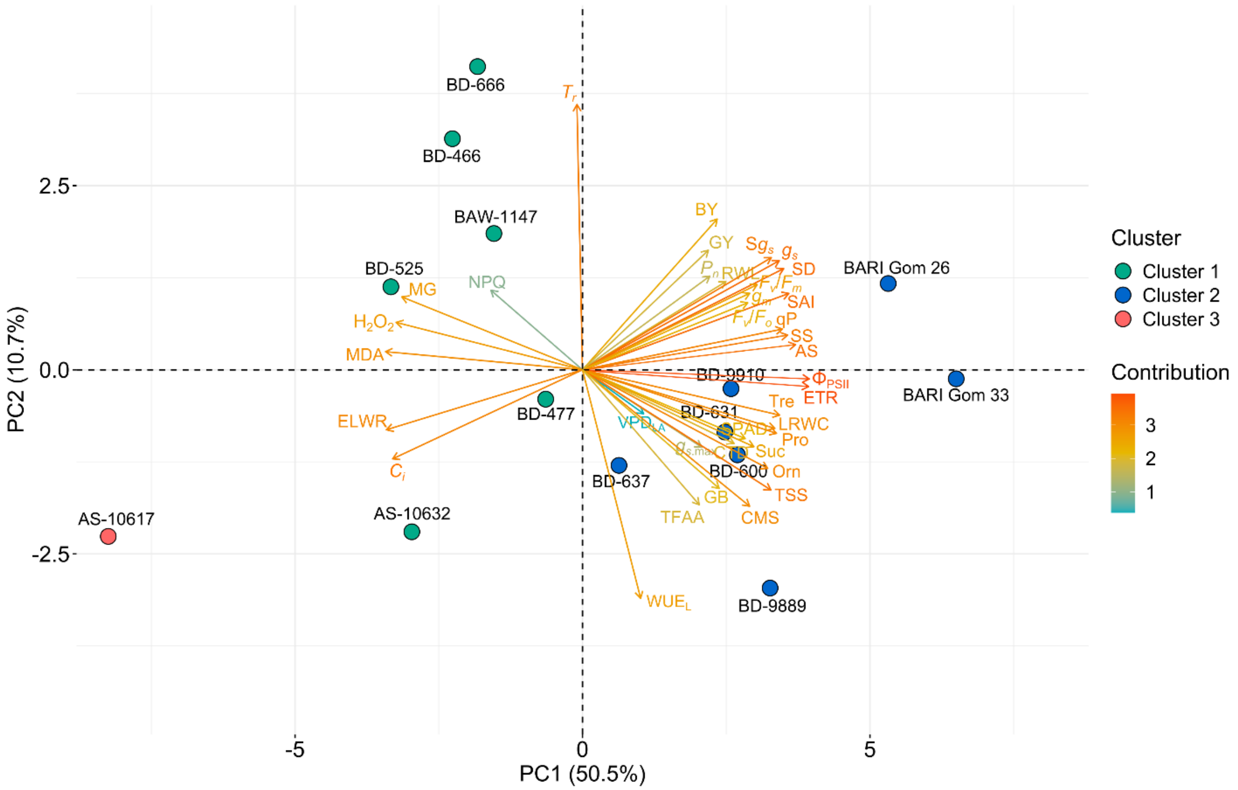

Figure S12. PCA-Biplot of wheat genotypes and studied traits constructed from relative trait values. Based on their dissimilarity, genotypes were distributed in distinct ordinates. In the biplot, the length indicates the quality of representation and the color intensity of a vector represents the contribution of the traits to the principal components. Positive or negative interactions of examined parameters are shown by the angles between the vectors produced from the center point of biplots. Additional details are shown in Table S4.

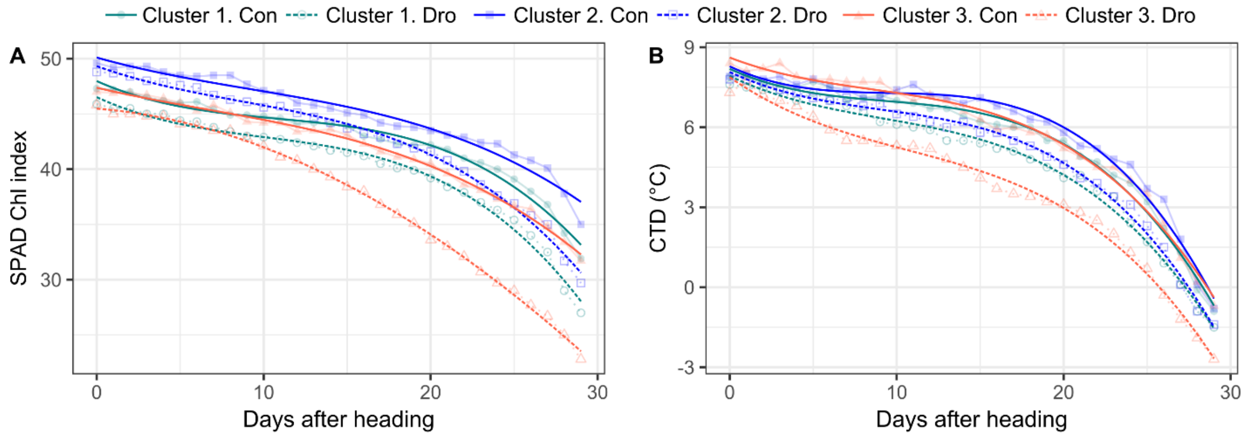

Figure S13. Cluster-wise changes in SPAD chlorophyll index (A) and canopy temperature depression [CTD] (B) of the wheat genotypes measured everyday after heading under control and drought stress.

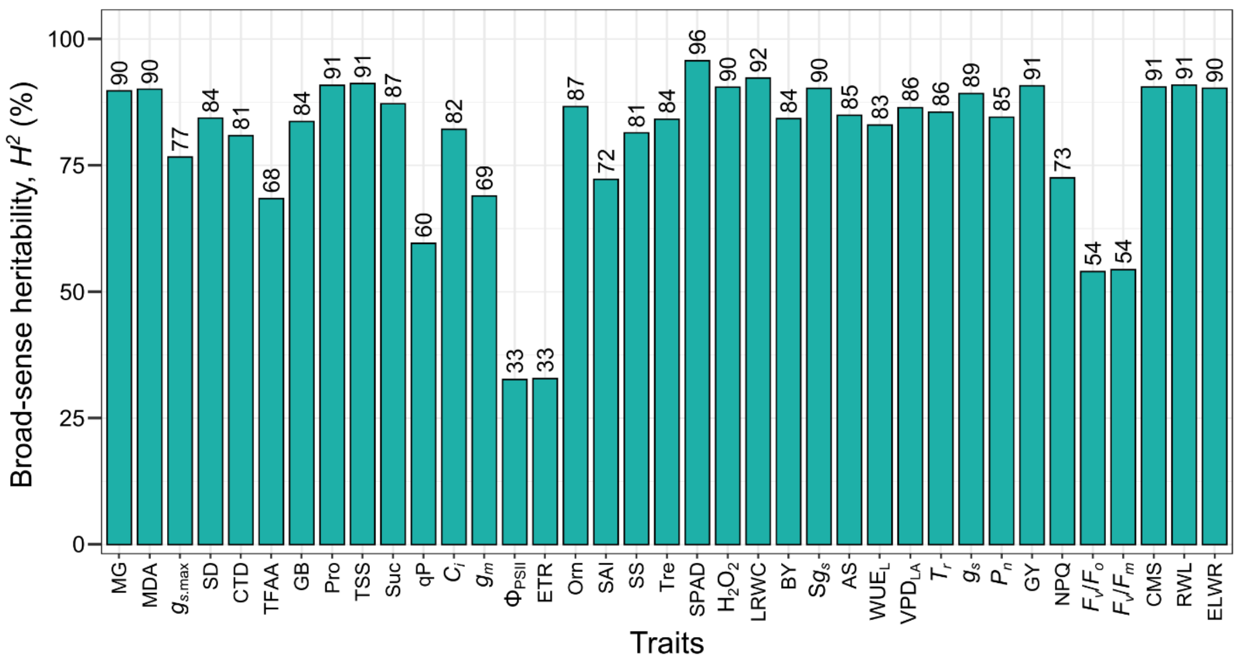

Figure S14. Broad-sense heritability (H^2) of the studied traits across the growing environments. Additional details are shown in Table S4.
